# Active vision is linked to category selectivity in the individual brain

**DOI:** 10.1038/s41562-026-02494-5

**Published:** 2026-07-07

**Authors:** Diana Kollenda, Elaheh Akbari, Maximilian D. Broda, Benjamin de Haas

**Affiliations:** 1 Experimental Psychology, Justus-Liebig University Giessen, Giessen, Germany; 2 Center for Mind, Brain and Behaviour, Marburg and Giessen, Germany

**Keywords:** Human behaviour, Visual system

## Abstract

Individuals reliably differ in how they look at complex visual scenes, with the most prominent variation in their propensity to fixate faces and text. Here we tested the hypothesis that these differences in gaze are linked to representational properties of the individual visual system in 61 adults. Eye-tracking captured each observer’s characteristic gaze tendencies during naturalistic scene viewing, and independent functional magnetic resonance imaging recorded category-selective responses to faces, words and other stimuli when participants were instructed to fixate centrally. We find that the propensity to fixate faces or text goes along with enhanced distinctiveness and enlarged functional regions of corresponding categorical representations in the ventral stream. These in turn predicted performance on reading and face recognition tasks. Thus, active vision appears linked to the precision of category-selective encoding and corresponding neural resources in the individual brain.

## Abstract (web summary)

In this Article, Kollenda et al. find that individual differences in gaze are linked to neural tuning in individual brains: stronger tendencies to look at faces or text correlate with more distinct neural representations of the corresponding category.

## Main

Vision is an active process that requires constant eye movements to explore different points of interest with high acuity. Typically, this process is modelled on the basis of group averages^1–3^. However, recent findings have documented robust differences between individuals viewing the same complex scenes, most prominently in the propensity to fixate faces and text^4–7^. These gaze preferences are linked to a tendency to prioritize corresponding elements when describing a scene^8^ and, at least for faces, to category-specific recognition skills^5,9^. Despite the striking effect sizes of individual differences in gaze^5,7^ and their known genetic component^10,11^, the underlying biological mechanisms are unclear. Why do some individuals fixate faces two to three times more often than other observers, even when viewing identical stimuli? Why does the same hold for the tendency to fixate text?

Several lines of research suggest that face and text fixation preferences may be linked to representational properties of the ventral stream. Just as individual differences in the functional anatomy of early visual cortex predict low-level perceptual and psychophysical biases^12,13^, face recognition performance correlates with the degree of face selectivity and the size of the right fusiform face (rFFA) area^14,15^. Moreover, individual differences in the tendency to fixate faces and text while watching movie clips predict interindividual representational divergence in the inferior temporal cortex^16^. Generally, for adult readers, faces and text are both the most salient types of fixation targets during scene viewing^5,17^ and the most prominent drivers of neural activity in distinct patches of the ventral stream^18–20^. Crucially, recent data from non-human primates show strongly reduced face fixation preferences in animals that lack ventral face patches, either naturally^21^ or due to experimentally induced developmental face deprivation^22^. Interestingly, the animals in these studies also showed an increased tendency to fixate hands, which, at least in Arcaro et al.’s.^22^ study, went along with increased neural selectivity for hands in the typical anatomical location of face patches. This resonates with recent human studies that suggest a developmental push–pull relationship between ventral selectivity for faces and hands^23^. Further, recent developmental studies have documented that the unfolding of text and face salience is protracted well into adolescence^24,25^, which parallels the development of corresponding ventral category representations^23,26–29^. Specifically, Nordt et al.^26^ used a measure of representational distinctiveness, which captures both the reliability and specificity of multivariate activation patterns evoked by a given stimulus category. Their data show that the distinctiveness of word and face representations continuously increases into adolescence, in parallel to reading and face recognition skills. This is strikingly similar to the findings of the large-scale eye-tracking study by Linka et al.^25^, who found that the proportion of immediate saccades towards faces and text during scene viewing continuously increases from age 5 years to at least 16 years.

These parallel developmental trajectories raise the possibility of a systematic link between active vision and representational spaces. Specifically, gaze tendencies may follow neural development, actively prioritizing semantic categories that can be represented more distinctively. Vice versa, developing gaze tendencies and changes in visual diet may shape representational distinctiveness. These hypotheses are not mutually exclusive and share a straightforward prediction: if there is a systematic link between gaze and representational distinctiveness, large individual differences in gaze tendencies among adults should correlate with corresponding category distinctiveness in the individual brain. To test these predictions, we quantified the individual representational distinctiveness of faces and words^26^ in the ventral temporal cortex (VTC) of 61 adult observers for whom we also quantified corresponding gaze tendencies in an independent free-viewing experiment^5,7^.

## Results

### Replication of stable individual gaze tendencies towards faces and text

**Fig. 1:**
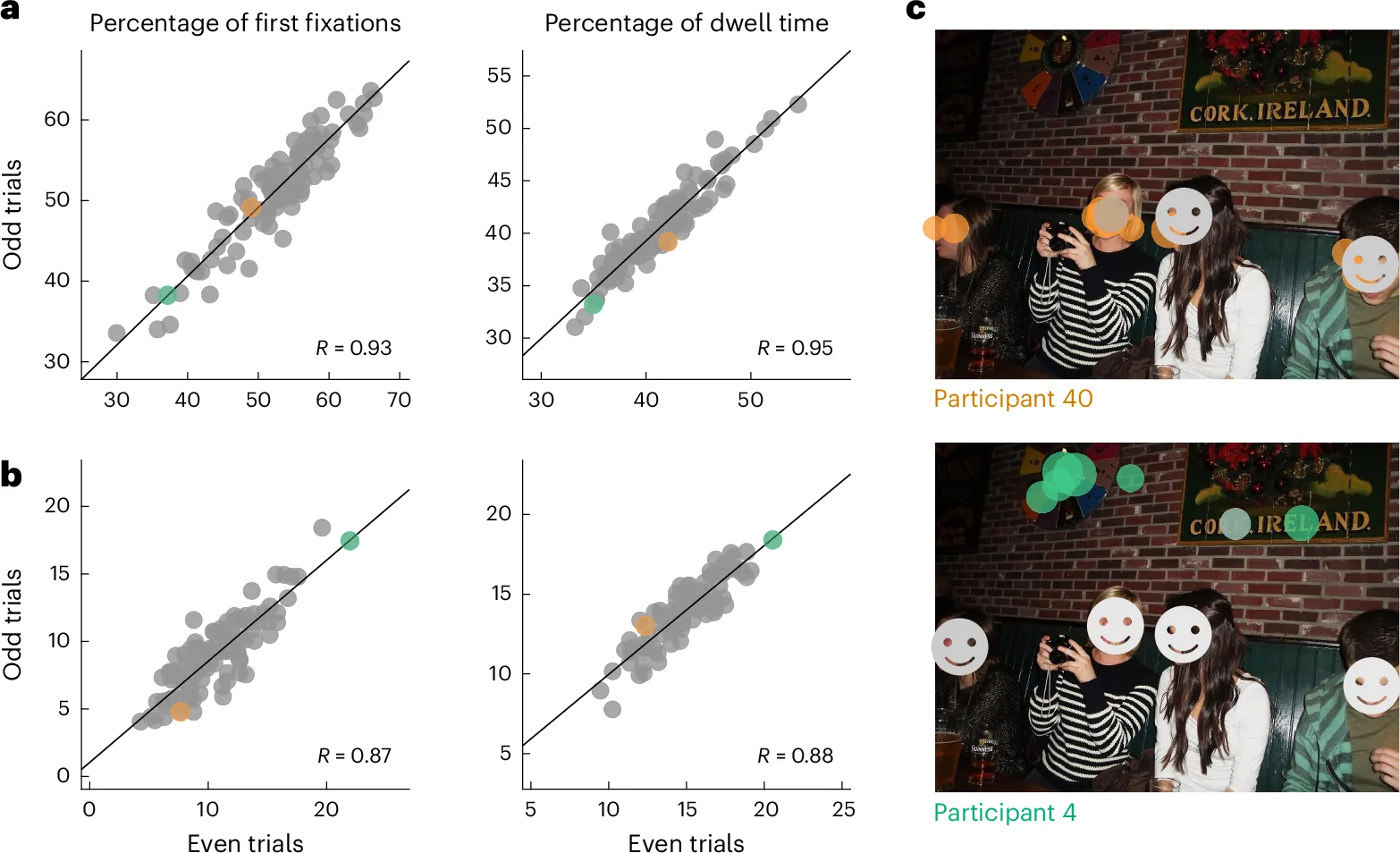
Consistent gaze tendencies towards faces and text in complex scenes. **a**,**b**, The consistency of individual proportions of first fixations and dwell time on faces (**a**) and text (**b**) across odd and even images (*N* = 102, all *r* > 0.87, *P* < 0.001). Coloured dots highlight data from participant ID4 in green and participant ID40 in orange. **c**, Corresponding gaze patterns for an example scene are visualized. Lighter colours represent first fixations and darker shades later ones. Disk sizes scale according to fixation durations. Faces were visible in the experiment but are obscured in this reproduction to protect privacy. Image in **c** from the OSIE dataset of Xu et al.^2^, published under an MIT license, ©2014 NUS VIP–Visual Information Processing Lab.

Gaze tendencies towards faces and text have been shown to be consistent across hundreds of complex scenes as well as across time^5,7^. This is true not only for the total duration spent on a given semantic category (that is, cumulative dwell time) but also for the tendency to target a given category with the first saccade after image onset. Here, we replicate these findings in 102 participants. For the individual proportion of first fixations on faces, we found excellent split-half reliability across odd and even images, *r*(100) = 0.93, *P* < 0.001, 95% confidence interval (CI) 0.90 to 0.95 (maximum–minimum ratio of 2.04). The same was true for the individual proportion of cumulative dwell time on faces, *r*(100) = 0.95, *P* < 0.001, 95% CI 0.92 to 0.97 (maximum–minimum ratio of 1.66). Similarly, we found high split-half reliability for the individual proportion of first fixations on text elements, *r*(100) = 0.87, *P* < 0.001, 95% CI 0.80 to 0.91 (maximum–minimum ratio of 4.71), as well as for the proportion of cumulative dwell time on text elements, *r*(100) = 0.88, *P* < 0.001, 95% CI 0.84 to 0.91 (maximum–minimum ratio of 2.16). In line with previous studies, there was a strong anti-correlation between the individual tendency to fixate faces or text (first fixations, *r*(100) = −0.50, *P* < 0.001, 95% CI −0.64 to −0.34; dwell time, *r*(100) = −0.52, *P* < 0.001, 95% CI −0.65 to −0.36). However, these anticorrelations were weaker when excluding the 175/700 images containing both faces and text, which put them in direct competition for attention (first fixations, *r*(100) = −0.15, *P* = 0.136, CI −0.33 to 0.05, dwell time, *r*(100) = −0.27, *P* = 0.006, 95% CI −0.44 to −0.09). Taken together, we could replicate large and consistent individual differences in the tendency to fixate faces and text (Fig. 1), which are strongly anticorrelated when brought into direct competition for attention.

### Representational distinctiveness is linked to individual gaze tendencies

**Fig. 2:**
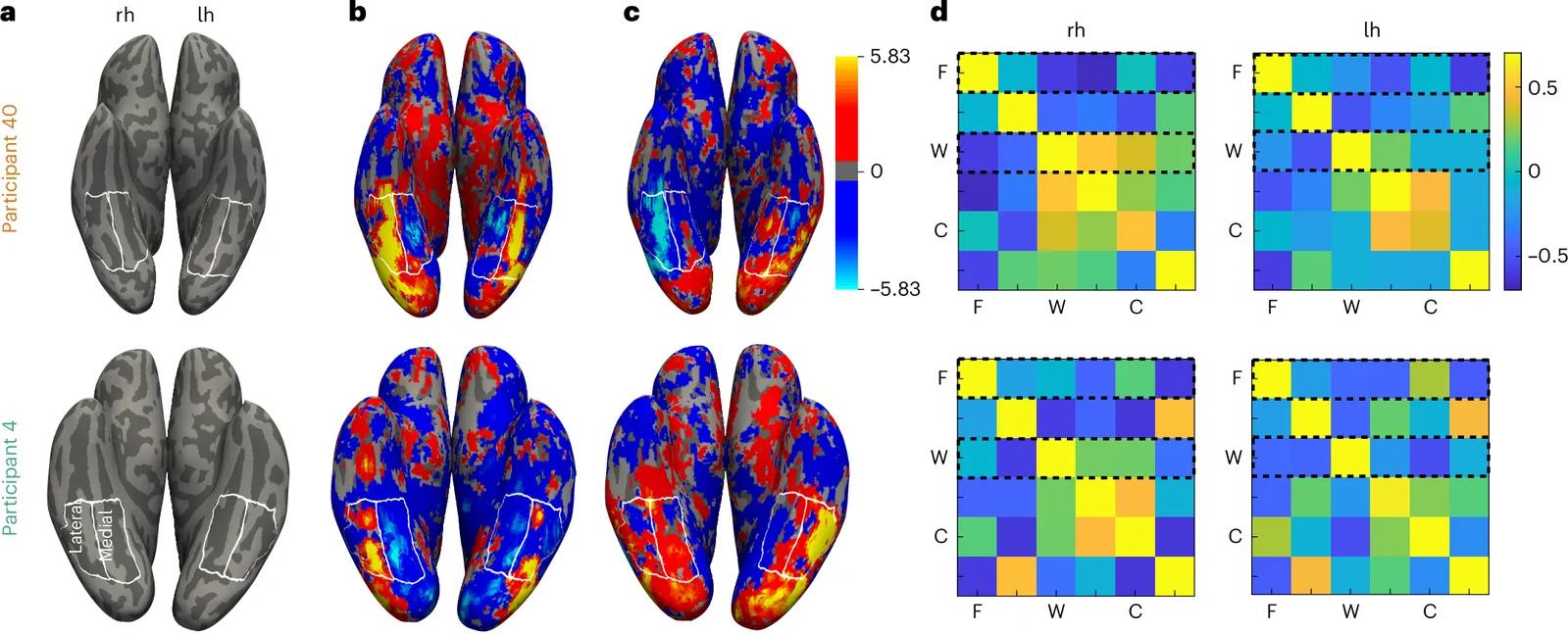
Multivariate pattern responses of individual participants in right and left lateral VTC. **a**, White outlines show individual anatomical ROIs for the lateral and medial VTC, in the left (lh) and right hemisphere (rh), based on anatomical landmarks following Nordt et al.^26^. **b**,**c**, Overlays of *t*-contrasts for face- (**b**) and word-specific (**c**) activations according to the inset colour bar on the right. **d**, RSMs for six stimulus categories including faces (F), words (W) and cars (C) in the lateral VTC, showing pattern-correlations according to the inset colour bar on the right. Rows used to calculate the distinctiveness of face and word representations are highlighted with dashed lines (within-category similarity in odd and even runs, that is, on diagonal values, minus the mean of between-category similarity in odd and even runs, that is, off-diagonal values). All panels show data from example participants ID4 (bottom) and ID40 (top).

In a separate session, we tested 61 of our eye-tracking participants in a standard functional localizer experiment in a magnetic resonance imaging scanner (subsequently abbreviated as functional magnetic resonance imaging (fMRI) session). This allowed us to identify specific patterns of neural activity associated with different object categories in the VTC of each individual. These categories included faces and pseudowords/characters along with a number of control categories (six categories in total: faces, headless bodies, pseudowords, houses, cars and limbs; compare (cf.) ^30^). Following Nordt et al.^26^, we determined the distinctiveness of the pattern evoked by a given category as the subtraction of average between-category correlations from within-category correlations (with the latter corresponding to pattern reliability; see Fig. 2 and Methods for more details). This measure of pattern distinctiveness has a theoretical range of −2 to 2, with higher values indicating more distinctive representations.

We calculated category distinctiveness separately for both hemispheres within individually defined regions of interest (ROIs) of the lateral VTC, following the anatomical definition by Nordt et al.^26^ (cf. ^18^). Lateral VTC is associated with a centre-biased recognition of form, objects and categories such as faces^31^ and words/characters^29^, whereas the medial part is associated with a periphery bias and selectivity to places and scenes (cf. ^30,32,33^). Notably, there is a hemispheric lateralization, with word-selective regions predominantly in the left hemisphere and face-selective regions primarily in the right^34^. We replicated this lateralization pattern in our sample but found no association between the individual degree of lateralization and gaze or perceptual performance (Supplementary Methods and Results).

The individual degree of representational distinctiveness for faces and words varied considerably. Face distinctiveness in the right lateral VTC exhibited a maximum–minimum ratio of 1.38 across participants. Word distinctiveness in the left lateral VTC showed a maximum–minimum ratio of 1.52 across participants.

**Fig. 3:**
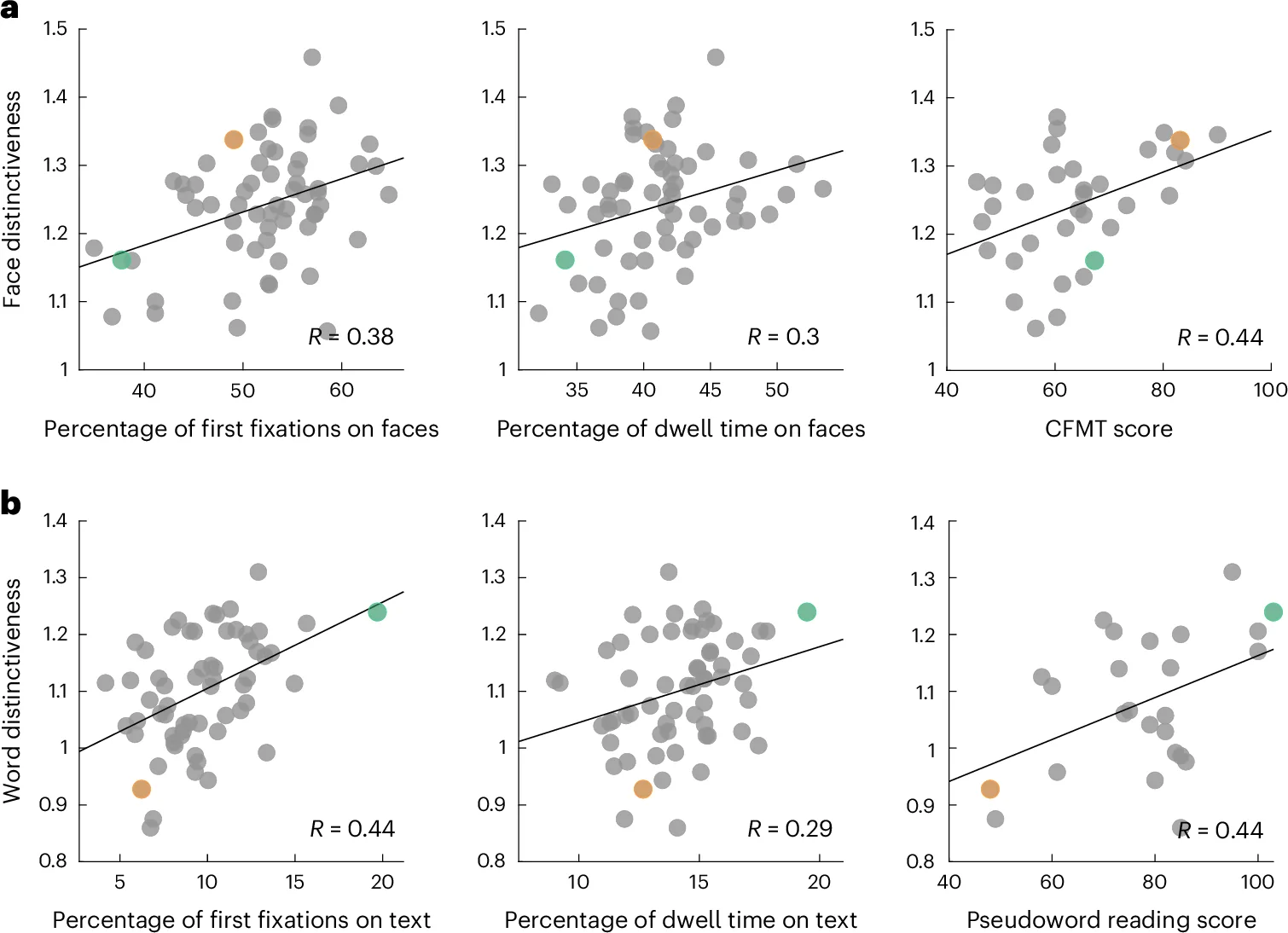
Category distinctiveness in right and left lateral VTC is linked to gaze tendencies and behavioural performance. **a**, Pearson correlations between face distinctiveness in right lateral VTC and the proportion of first fixations on faces (*N* = 61, *P* = 0.003), the proportion of dwell time on faces (*N* = 61, *P* = 0.017) and the CFMT score (*N* = 34, one-tailed *P* = 0.004), respectively. **b**, Pearson correlations between word distinctiveness in left lateral VTC and the proportion of first fixations on text (*N* = 61, *P* < 0.001), the proportion of dwell time on text (*N* = 61, *P* = 0.023) and the pseudoword reading score (*N* = 25, one-tailed *P* = 0.012), respectively. The coloured dots highlight the same individuals across figures (participant ID4 in green and participant ID40 in orange).

Next, we tested two primary a priori hypotheses: first, the individual tendency to fixate faces is linked to face distinctiveness in the right lateral VTC and second, the individual tendency to fixate text is linked to word distinctiveness in the left lateral VTC. We quantified category-specific fixation tendencies as the proportions of first fixations and dwell time spent on corresponding scene elements in the free-viewing experiment (and applied Bonferroni-corrected significance thresholds of *α* = 0.025 to account for the use of these two measures). Crucially, face distinctiveness in right lateral VTC significantly correlated with the tendency to direct first fixations (*r*(59) = 0.38, *P* = 0.003, 95% CI 0.15 to 0.56) and dwell time (*r*(59) = 0.30, *P* = 0.017, 95% CI 0.13 to 0.47) to faces in the independent free-viewing experiment (Fig. 3a; see also Supplementary Table 1 for an overview of all correlations). Similarly, word distinctiveness in the left lateral VTC was correlated with the tendency to direct first fixations (*r*(59) = 0.44, *P* < 0.001, 95% CI 0.25 to 0.60) and dwell time (*r*(59) = 0.29, *P* = 0.023, 95% CI 0.08 to 0.49) on text (Fig. 3b).

Furthermore, we explored whether the observed relationships between neural and gaze patterns are hemisphere-specific. Word distinctiveness in right lateral VTC was positively correlated with the tendency to direct first fixations on text (*r*(59) = 0.35, *P* = 0.006, 95% CI 0.13 to 0.55) but not dwell time (*r*(59) = 0.14, *P* = 0.299, 95% CI −0.05 to 0.32). There were no significant correlations between face distinctiveness in left lateral VTC and gaze tendencies towards faces (first fixation and dwell time both *P*(59) >0.12; see Supplementary Table 1 for detailed results). An exploratory whole-brain searchlight analysis replicated associations between gaze tendencies and corresponding category distinctiveness in VTC. Moreover, we found that the tendency to direct first fixations to text correlated with word distinctiveness in regions of the extended reading network, especially in the left hemisphere (cf. ^35,36^; see Supplementary Methods and Results, including Supplementary Figs. 1 and 2, for more details).

Reassuringly, the observed correlations between representational distinctiveness and gaze were category-specific. Cross-correlations between word distinctiveness in left lateral VTC and gaze tendencies towards faces (first fixations and dwell time) were nonsignificant, both *P*(59) >0.22 (see Supplementary Table 1 for detailed results). Interestingly, face distinctiveness in right lateral VTC correlated negatively with the tendency to direct first fixations to text (*r*(59) = −0.36, *P* = 0.005, 95% CI −0.55 to −0.14), whereas dwell time on text did not (*r*(59) = −0.24, *P* = 0.058, 95% CI −0.43 to −0.04, not significant).

Together, these findings show a robust and specific link between individual gaze tendencies towards faces and text and the distinctiveness of corresponding representations in the individual ventral stream.

### Representational distinctiveness is linked to behavioural performance

Nordt et al.^26^ reported that word distinctiveness in left and face distinctiveness in right lateral VTC predicts reading and face recognition performance across children and adolescents. To test whether this relationship extends to individual differences in our adult sample, we invited participants back for additional testing and were able to do so in subsamples. Specifically, we examined in 34 participants whether face distinctiveness in the right lateral VTC correlates with face recognition ability measured with the Cambridge Face Memory Test (CFMT) in the long format^37^. In line with prior developmental findings^26^, we observed a positive correlation between face distinctiveness in right lateral VTC and the CFMT score, *r*(32) = 0.44, one-tailed *P* = 0.004, 95% CI 0.20 to 0.64 (Fig. 3a, right-most plot; Methods). Furthermore, we investigated whether word distinctiveness in left lateral VTC is linked to reading performance, measured with a speed-reading test for words and pseudowords (Salzburger Lese- und Rechtschreibtests (SLRT-2))^38^ in a subsample of 25 participants. Here, we observed a positive correlation between word distinctiveness in left lateral VTC and the reading-speed scores for pseudowords *r*(23) = 0.44, one-tailed *P* = 0.012, 95% CI 0.03 to 0.72 (Fig. 3b, right-most plot; Methods). This also is in line with the previous study by Nordt et al.^26^. There was no significant correlation between word distinctiveness in left lateral VTC and reading-speed scores for words, *r*(23) = 0.17, one-tailed *P* = 0.204, 95% CI −0.30 to 0.62.

### The individual sizes of rFFA and lVWFA are linked to gaze tendencies and behavioural performance

Our previous analyses focused on the multivariate measure of category distinctiveness within anatomically defined ROIs, which develops in tandem to category-specific salience (cf. ^25,26^). However, many previous studies used univariate measures of face and word-selectivity (cf. ^23,29,30^). Everything else staying equal, both types of measures are expected to scale with the signal-to-noise ratio of category-specific responses. However, in principle, they may also vary independently. While distinctiveness probes the sharpness of (potentially distributed) category-specific response patterns at the population level, the extent of category-specific responses probes the amount of neural resources dedicated to processing a given category. Here, we sought to test whether individual gaze tendencies and perceptual performance are also linked to univariate category selectivity, specifically the individual sizes of the rFFA and left visual word form area (lVWFA).

To this end, we quantified the participant-specific selective activation within group-level parcels for the rFFA and lVWFA (cf. Julian et al.^39^; see the Methods for details). We observed high split-half reliability (odd versus even localizer runs) for interindividual differences in the sizes of these functionally defined ROIs (rFFA, *r*(59) = 0.92, *P* < 0.001, 95% CI 0.88 to 0.95; lVWFA, *r*(59) = 0.82, *P* < 0.001, 95% CI 0.73 to 0.89). See Supplementary Table 1 for an overview of all tested correlations with functional ROI (fROI) sizes.

**Fig. 4:**
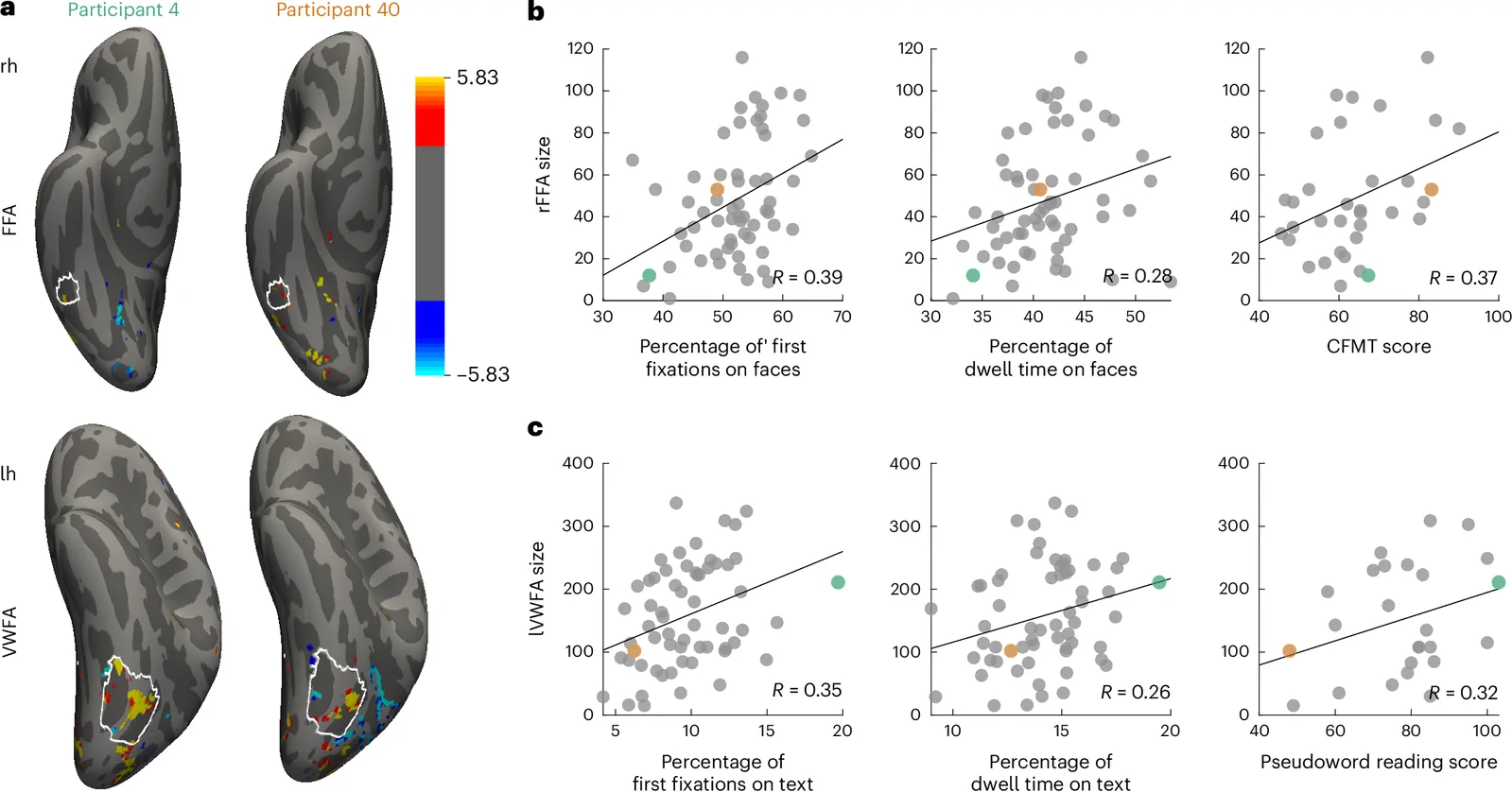
The relationship between the individual size of rFFA and lVWFA, gaze tendencies and behavioural performance. **a**, Top: the outline of the group-level rFFA parcel in MNI space overlaid with individual *t*-contrasts for face-specific activations, ranging from |*t*| > 4.28 (*P* < 0.0001) to 5.83, as indicated by the colour bar to the right. Bottom: similarly, group-level parcels for the lVWFA overlaid with individual *t*-contrasts for word-specific activations. **b**, Pearson correlations between the individual size of rFFA and the proportion of first fixations on faces (*N* = 61, *P* = 0.002), the proportion of dwell time on faces (*N* = 61, *P* = 0.031, not significant (n.s.), Bonferroni-corrected) and the CFMT score (*N* = 34, one-tailed *P* = 0.014), respectively. **c**, Pearson correlations between the individual size of lVWFA and the proportion of first fixations on text (*N* = 61, *P* = 0.006), the proportion of dwell time on text (*N* = 61, *P* = 0.039, n.s., Bonferroni-corrected) and the pseudoword reading score (*N* = 25, one-tailed *P* = 0.060, n.s.), respectively. Coloured dots highlight the same individuals across figures (participant ID4 in green and participant ID40 in orange). lh, left hemisphere; rh, right hemisphere; FFA, fusiform face area; VWFA, visual word form area.

Individual sizes of rFFA correlated positively with the tendency to first fixate on faces (*r*(59) = 0.39, *P* = 0.002, 95% CI 0.17 to 0.58) but not with the tendency of higher dwell times on faces (*r*(59) = 0.28, *P* = 0.031, 95% CI 0.02 to 0.52, not significant at the Bonferroni-corrected threshold of *α* = 0.025). Individual rFFA sizes were negatively correlated with gaze tendencies towards text (first fixations, *r*(59) = −37, *P* = 0.004, 95% CI −0.56 to −0.13; dwell time, *r*(59) = −0.34, *P* = 0.007, 95% CI −0.52 to −0.13). Furthermore, rFFA sizes correlated positively with face distinctiveness in the right lateral VTC (*r*(59) = 0.55, *P* < 0.001, 95% CI 0.36 to 0.70) and with behavioural performance on the CFMT (*N* = 34; *r*(32) = 0.37, one-tailed *P* = 0.014, 95% CI 0.09 to 0.60; see also Fig. 4b and Methods for more details). The latter result aligns with previous findings by Furl et al.^14^ and Elbich and Scherf^15^.

Individual sizes of lVWFA correlated positively with the tendency to first fixate text (*r*(59) = 0.35, *P* = 0.006, 95% CI 0.15 to 0.54) but not with dwell time on text (*r*(59) = 0.26, *P* = 0.039, 95% CI 0.06 to 0.45, not significant at the Bonferroni-corrected threshold of *α* = 0.025). No significant cross-correlations were observed for gaze tendencies towards faces (first fixations, *r*(59) = 0.09, *P* = 0.514, 95% CI −0.14 to 0.31; dwell time, *r*(59) = 0.01, *P* = 0.962, 95% CI −0.21 to 0.21). lVWFA sizes showed a strong positive correlation with word distinctiveness in the left lateral VTC (*r*(59) = 0.75, *P* < 0.001, 95% CI 0.63 to 0.83). However, the correlation between lVWFA sizes and reading-speed scores for pseudowords did not reach significance (*N* = 25; *r*(23) = 0.32, one-tailed *P* = 0.060, 95% CI −0.05 to 0.59; Fig. 4c, Methods and Supplementary Table 1), which may reflect the small sample size, especially in light of previous reports of significant correlations between lVWFA size and reading scores^40^.

Overall, these results mirror the patterns observed between gaze tendencies, category distinctiveness and behaviour (Fig. 3 and Supplementary Table 1). They suggest that active vision is linked to both: the sharpness of neural tuning for and resources dedicated to processing a given category in the individual brain. Our results are also consistent with the previous developmental finding that increases in category selectivity go along with increases in category distinctiveness (cf. ^26^). However, in contrast to distinctiveness, category-specific associations between area sizes and dwell time as well as reading scores were not significant.

### No support for a link between gaze tendencies and elevated levels of attention during the localizer task

The link between gaze tendencies and category-selective activity in VTC could in principle be mediated by category-specific levels of attention, generalizing between free-viewing and the localizer task. Specifically, participants with a stronger tendency to fixate faces or words during free-viewing may also have allocated higher levels of attention to the respective type of stimulus when it was presented centrally and in isolation during the fMRI localizer task. This hypothesis would align with the finding that experts direct more attention to their object of expertise^41–43^, potentially leading to increased activation levels in VTC^44–47^.

To test this hypothesis, we conducted an additional psychophysics experiment in 60 naive adults who also completed an abbreviated but sensitive version of the free-viewing experiment (200 images; cf. Linka and de Haas^7^). Crucially, the new psychophysics experiment mirrored our fMRI localizer design but introduced a secondary blob-detection task to probe differential levels of attentional competition with faces or pseudowords (cf. ^48^, and see the Methods for details). This design enabled two key tests: first, to determine whether there are systematic individual differences in the level of attentional competition afforded by isolated, centrally presented faces and pseudowords, and second, to test whether these differences in attention correlate with the tendency to fixate faces or words during free-viewing. While a positive correlation would support the mediation account, the absence of a detectable correlation would speak against attentional engagement mediating the observed link between gaze tendencies and category-selective activity in VTC.

First, participants indeed showed reliable individual differences in secondary task performance for face blocks (*M* = 73%, s.d. of 10.2%; split-half reliability *r*(58) = 0.66, *P* < 0.001, 95% CI 0.45 to 0.80), pseudoword blocks (*M* = 85%, s.d. of 17.2%; split-half reliability *r*(58) = 0.92, *P* < 0.001, 95% CI 0.58 to 0.97) and the performance difference between them (face-block advantage *M* = 11.7% s.d. of 16.6%; split-half reliability *r*(58) = 0.81, *P* < 0.001, 95% CI 0.05 to 0.93). Second, however, we found no significant associations between this indicator of category-specific differential levels of attention and gaze tendencies towards faces (first fixations, *r*(58) = −0.03, *P* = 0.814, BF_10_ = 0.17, 95% CI −0.20 to 0.16; dwell time, *r*(58) = 0.08, *P* = 0.528, BF_10_ = 0.20, 95% CI −0.19 to 0.32) or text (first fixations, *r*(58) = −0.03, *P* = 0.844, BF_10_ = 0.16, 95% CI −0.19 to 0.24; dwell time, *r*(58) = −0.01, *P* = 0.951, BF_10_ = 0.16, 95% CI −0.14 to 0.24). This suggests that the relationship between gaze and category-selective activity in VTC is unlikely to be mediated by elevated levels of category-specific attention.

### Immediate face salience is linked to face recognition performance

**Fig. 5:**
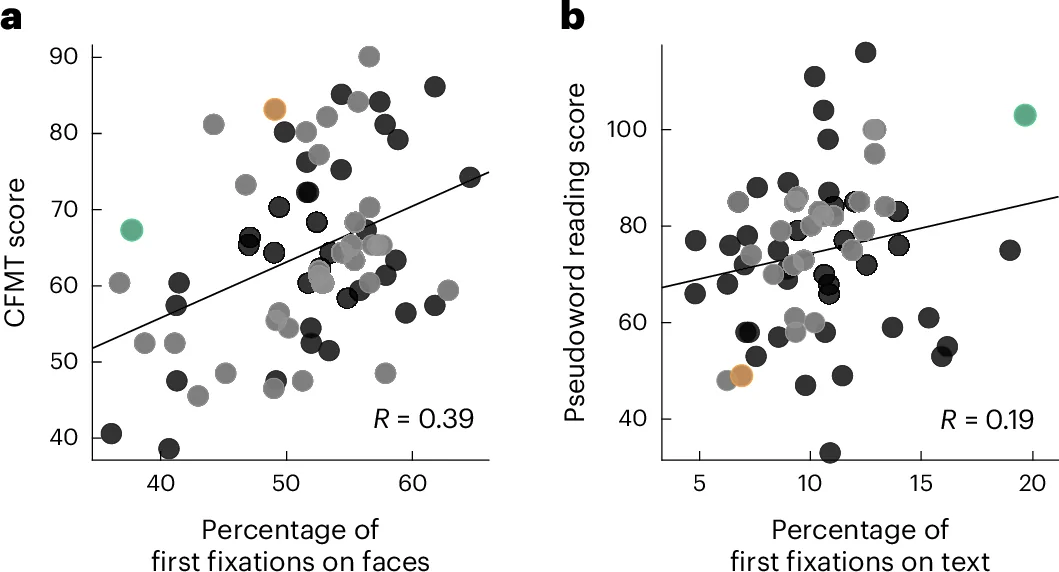
Proportions of first fixations on faces are related to face recognition, whereas first fixations on text are not significantly related to reading performance. **a**,**b**, The Pearson correlation between the proportion of first fixations on faces and CFMT scores (*N* = 69, *P* < 0.001) (**a**), as well as first fixations on text and pseudoword reading scores (*N* = 65, *P* = 0.065, not significant)(**b**). In both plots, black dots represent participants from the eye-tracking experiment only, and light grey dots indicate those who also participated in the fMRI session. Coloured dots highlight the same individuals across figures (participant ID4 in green and participant ID40 in orange).

The CFMT performance scores showed considerable variability, with a maximum–minimum ratio of 1.98 among the 34 fMRI participants (*M* = 64.08, s.d. of 11.87) and 2.33 for an extended sample of 69 participants (*M* = 64.37, s.d. of 11.95), including participants who took part in the free-viewing experiment and CFMT but not the fMRI session. In the extended sample, a significant positive correlation emerged between the proportion of first fixations on faces and CFMT scores, *r*(67) = 0.39, one-tailed *P* < 0.001, 95% CI 0.17 to 0.58. This relationship was not significant within the fMRI subsample (*r*(32) = 0.23, one-tailed *P* = 0.094, 95% CI −0.07 to 0.49, not significant; Fig. 5a). In line with previous results^5^, we observed no significant correlation between dwell times on faces and CFMT performance in both the extended sample (*r*(67) = 0.18, one-tailed *P* = 0.077, 95% CI −0.04 to 0.38, not significant) and the smaller fMRI sample (*r*(32) = 0.16, one-tailed *P* = 0.174, 95% CI −0.17 to 0.49, not significant). These findings replicate those of de Haas et al.^5^, who reported an association between CFMT scores and the tendency to direct first fixations to faces but not dwell times.

Performance on the speed-reading test showed considerable variability across the 25 fMRI participants and an extended sample of 65 participants for both words (subsample, *M* = 118.40, s.d. of 16.17, maximum–minimum ratio of 1.97; extended sample, *M* = 111.35, s.d. of 18.07, maximum–minimum ratio of 2.11) and pseudowords (subsample, *M* = 77.92, s.d. of 14.57, maximum–minimum ratio of 2.15; extended sample, *M* = 74.68, s.d. of 16.36, maximum–minimum ratio of 3.52). Word and pseudoword performance were positively correlated in the extended sample, *r*(63) = 0.65, *P* < 0.001, 95% CI 0.50 to 0.78. Among the fMRI participants, we observed significant positive correlations between the proportion of first fixations on text in complex scenes and speed-reading test scores for both words (*r*(23) = 0.50, one-tailed *P* = 0.004, 95% CI 0.23 to 0.74) and pseudowords (*r*(23) = 0.69, one-tailed *P* < 0.001, 95% CI 0.43 to 0.84). However, in the extended sample, this relationship vanished for words (*r*(63) = −0.04, one-tailed *P* = 0.386, 95% CI −0.31 to 0.24) and pseudowords (*r*(63) = 0.19, one-tailed *P* = 0.065, 95% CI −0.04 to 0.42, not significant; Fig. 5b). No significant correlations were observed between the proportion of dwell time on text and both reading performances (Supplementary Table 1). Although this renders the evidence regarding a potential relationship between gaze tendencies and reading performance inconclusive, very strong effect sizes seem unlikely, given the high internal consistency of both the gaze (see above) and performance measures (cf. ^38^).

Taken together, our results show that category-specific differences in behavioural performance among adults are linked to individual representational distinctiveness (cf. ^26^) and, at least for faces, also to the size of the rFFA and immediate category-specific salience (cf. ^5,9^). However, given that these experiments were limited to small subsamples available for behavioural testing, they should be interpreted with caution.

## Discussion

Across fMRI and eye-tracking, our findings show that the individual tendency to fixate faces or text targets elements of a scene that can be represented more distinctively in the individual visual system. Moreover, a complementary analysis showed that the individual tendency to immediately fixate faces or text also goes along with larger corresponding ROIs (rFFA and lVWFA, respectively).

This provides a link between individual biology and recently discovered large and systematic differences in gaze^5^. A previous study from our group demonstrated that individual gaze during movie watching goes along with concurrent representational differences in the ventral stream. Specifically, the authors showed that the interindividual similarity of neural representations during movie watching is determined by the similarity of gaze^16^. Here, we asked a different question: do neural representations as such differ between people with different gaze tendencies, even under conditions of central fixation? If so, how do neural representations differ between people with different gaze tendencies? We found that centrally presented faces and pseudowords are represented more distinctively in individuals who tend to fixate these objects when they are embedded in complex scenes. Moreover, the brains of individuals prioritizing faces or text had larger corresponding ROIs in the ventral stream (rFFA and lVWFA, respectively). This suggests that gaze prioritizes retinal input that the individual brain can process more distinctively.

Furthermore, we found that stronger face recognition and reading performance correlated with greater corresponding representational distinctiveness in the lateral VTC. These results extend previous findings on neurodevelopmental^26^ and individual^14^ links between categorical tuning in the ventral stream and perceptual performance. We also replicated a correlation between the tendency to initially fixate faces and better face recognition performance^5^.

The links between gaze tendencies, neural selectivity and perceptual performance were overall stronger for first fixations, that is, the tendency to immediately fixate corresponding elements in a scene. An intriguing distinction between first and subsequent fixations is that first fixations are often considered to be driven by automatic, bottom-up processes^49^, which tend to decrease as viewers freely and more deliberately explore visual scenes^50^. Interestingly, our results suggest that such bottom-up processes may be particularly aligned with category-selective processing in the individual VTC. Notably, Vinken and Vogels^21^ reported a pronounced reduction of face salience for early fixations in a monkey with lacking face patches. Furthermore, Sheinberg and Logothetis^51^ found that neurons in the temporal cortex of monkeys exhibited selective activation shortly before saccades to behaviourally relevant (that is, target) objects, and this activity raised again during fixation. Similarly, a preliminary report of intracranial recordings confirms presaccadic, category-specific VTC responses in humans^52^. Thus, although VTC traditionally is not considered part of the oculomotor system, it appears tightly linked to the control of gaze, perhaps by signalling object identity and relevance to downstream visuomotor networks (cf. ^53,54^). Future research is needed to clarify the nature and timing of this potential influence.

Importantly, our findings do not allow any direct conclusions regarding the developmental interplay of category distinctiveness, gaze tendencies and behavioural performance. These relationships may be modulated by individual visual experience (cf. a recent preprint by Yang et al.^55^), expertise in visual object recognition, attentional habits and genetic predispositions, which can shape perceptual biases and neural architecture (see below). Regardless of preceding developmental trajectories, our findings reveal an empirical association between moment-to-moment gaze behaviour and the overarching capability of more distinctive neural representation in the adult ventral stream. This broadly echoes theories of active inference^56^, which propose that individuals actively seek out observations reducing uncertainty given their internal representations of the world.

Why do active vision and neural tuning in the ventral stream match each other? Our main approach is correlational, but several additional observations lead us to an informed speculation: individual gaze and neural tuning in the ventral stream may mutually shape each other across the first two decades of life, whereas adults match their moment-by-moment gaze to stabilized tuning properties of the matured ventral stream.

The development of both gaze tendencies^25^ and neural tuning in the ventral stream^23,26,57^ is protracted across the first two decades of life. Developing fixation habits probably shape the individual foveal diet. Visual experience in turn can shape category-specific neural responses in the ventral stream, as shown for reading acquisition (for example, refs. ^27,29,58–60^), extensive exposure to Pokémon characters^61,62^ or face-deprivation in non-human primates^22^. At the same time, a lack of neural preferences goes along with a strongly reduced tendency to spontaneously fixate faces^21,22^, underscoring the possibility of a bidirectional link. Moreover, spontaneous gaze shows surprisingly strong genetic components^10,11^, which could partly be mediated by heritable differences in neural tuning^63,64^, including ventral stream preferences for faces^65,66^. Taken together, individual differences in gaze and neural tuning may mutually reinforce each other across development. However, this remains to be tested in multimodal longitudinal studies (see below).

In adults, both the individual layout of the ventral stream^67^ and individual differences in gaze^5^ appear stable. However, this stability seems more pronounced for neural tuning than gaze. Although attention and task instructions can modulate ventral stream responses top-down, this does not lead to a reversal of category-specific preferences in areas such as the fusiform face area^68^. Spontaneous gaze tendencies, by contrast, can be overridden and reversed top-down: even individuals with a strong tendency to look at faces manage to mostly look away from them if instructed to do so^69^. This suggests that, at least for adults, the moment-by-moment plasticity of gaze exceeds that of neural tuning in the ventral stream. Furthermore, individual differences in adult neural response patterns do not depend on concurrent differences in gaze. The results of our localizer experiment documented such differences under conditions of central fixation. Taken together, adults appear to show persistent individual differences in their neural preferences, which gaze caters to second by second. Future studies should test the causal nature of this link.

More generally, further research should trace neural and gaze development longitudinally to disentangle their interplay. Doing this in mono- and dizygotic twins would allow to differentiate between genetic and environmental effects^10,11^. Furthermore, studies utilizing perceptual training could manipulate the distinctiveness of individual representations to test their effect on gaze.

Theoretically, both gaze tendencies and neural preferences could be mediated by habitually elevated levels of covert and overt attention. If an individual has persistently elevated levels of attention for a given type of stimulus, this may boost corresponding neural responses and bias gaze in tandem. If true, this should also render the corresponding stimulus a more potent distractor in dual-task scenarios. Our additional psychophysical attention experiment was designed to put this prediction to a well-powered test. However, we found no such relationship, rendering persistent individual differences in category-specific levels of attention an unlikely mediating mechanism for the relationship between neural tuning and gaze.

Notably, expertise may play a role for both, salience and cortical selectivity (cf. ^61,62^). For instance, greater car expertise is associated with better car detection in scenes^43^, increased bottom-up salience for cars^70^ and increased VTC selectivity^44^. Given that performance differences on face recognition and reading tasks go along with corresponding category selectivity and gaze tendencies, our results can be interpreted along these lines. Developmentally, gaze tendencies, perceptual skills and neural tuning may reinforce each other (see above). This could be interpreted as a spiral of expertise, achieving different levels in different individuals.

Interestingly, however, expertise research found that widespread category-selective activity for objects of expertise diminished to novice-like responses when stimulus engagement was low^47^. Future studies could test whether high category-specific engagement tasks increase the size of the effects we observed here. This could be part of a broader endeavour to test how well individual differences in gaze, perceptual skills and neural tuning generalise between different types of tasks, including real-world behaviour. A first step in this direction is the observation of elevated face salience in super-recognizers, several of whom are employed for real-world face recognition tasks by the police^9^.

Notably, the observed correlations are based on a relatively homogeneous sample of healthy university students. Nevertheless, we observed striking individual variability: gaze tendencies varied substantially, with a 4.7-fold difference in the tendency to first fixate text elements and a 2-fold difference for faces. Neural representations also showed marked variation: word distinctiveness in the left lateral VTC differed by up to 1.5-fold and face distinctiveness in the right lateral VTC 1.3-fold. Similarly, category-specific recognition performance varied across individuals, with a 2.3-fold range in face recognition ability and up to 3.5-fold differences in reading speed. The degree of (co)variance in this homogeneous sample suggests that the observed effects are either relatively independent of age, level of education and general cognitive ability or could be even more extreme in more diverse samples. Recent developmental findings suggest the latter by documenting that gaze patterns in natural scenes tend to become less idiosyncratic and more canonical across childhood and adolescence^25^. Larger and more diverse samples could probe the role of multiple third variables and potential moderators such as gender, personality traits or academic-domain performance. However, the evidence for gender and personality associations with gaze tendencies is limited. For example, several previous studies observed a visual preference for social stimuli in females^71–73^, whereas recent large-scale eye-tracking work hast reported only very small gender differences in the opposite direction (Linka et al.^25^; *d* ≈ 0.2). Likewise, prior work has found no reliable link between personality traits such as extraversion and gaze tendencies (de Haas et al.^5^). Given the observed associations with reading scores, it seems especially important to investigate the developmental interplay of schooling, gaze tendencies, neural development and educational attainment.

In summary, our key finding is that individual differences in the tendency to fixate faces or text are systematically linked to corresponding neural resources and representational distinctiveness in the ventral stream. Previous research has shown that both individual gaze and the functional layout of the VTC undergo protracted and experience-dependent development. Our results show that in adulthood, active vision and neural tuning in the ventral stream are matched to each other. Moment-by-moment saccadic choices systematically prioritize elements of a scene which can be represented more distinctively in the individual visual brain.

## Methods

### Participants

We recruited a total of 102 healthy participants (mean age of 24.64 years, s.d. of 4.58 years; 30 male, 69 female, 3 diverse) from the participant mailing list at Justus-Liebig-University Giessen, Germany. All participants had normal or corrected-to-normal visual acuity and reported no known visual deficits. Every participant completed the eye-tracking experiment (~120 min). Additionally, a subset of participants took part in further tasks: 61 completed the fMRI session (~90 min), 69 completed the CFMT in the long format (~15 min, including 34 who also participated in the fMRI session) and 65, with German as their mother tongue, completed the speed-reading task (~5 min, including 25 who also participated in the fMRI session). Participants received either course credit or monetary compensation (10 euros per hour) for their participation. The study was approved by the local ethics committee of the Justus-Liebig-University Giessen (approval no. LEK-FB06) and was conducted in accordance with the relevant guidelines and regulations. All participants provided written informed consent.

### Procedure

Every participant took part in an eye-tracking session, followed by an fMRI session and/or a behavioural session. If participants took part in both the fMRI and behavioural sessions, the behavioural tests were typically completed after the scanning; otherwise, the CFMT was completed subsequently online using testable.org^74^. Before the eye-tracking session, fMRI session and behavioural tests, participants gave written informed consent and received task instructions. During the fMRI session, participants performed additional scans not related to the current study, including retinotopy, free viewing of 150 images and diffusion tensor imaging. Each session began with three localizer runs, followed by three retinotopy runs and then another set of three localizer runs.

### Eye-tracking

Stimuli were displayed and data collected using Psychtoolbox version 3.0.16^75^ and MATLAB (R2021b, MathWorks) on a Windows 10 computer and 23.8-inch LG Ultra HD28 monitor at a resolution of 3,840 pixels × 2,160 pixels. The display was placed at a distance of 55 cm, and stimuli were presented at a size of 2,400 pixels × 1,800 pixels, spanning 34.3 degrees of visual angle (dva) × 25.7 dva. Eye movements were recorded at a frequency of 1 kHz using an Eyelink 1000 (SR Research). Following the experimental design reported by de Haas et al.^5^, participants ran 700 trials in total, each showing one scene image from the OSIE image set^2^, which they could freely explore, and they were offered to take short breaks after every 100 trials. Each 100-trial block started with a nine-point calibration, followed by a validation. Calibration was accepted only if the maximum error was ≤1.2 dva and the average error was ≤0.6 dva. The observed mean validation error was 0.37 dva (s.d. of 0.11 dva). Participants initiated each trial by fixating on a central point while simultaneously pressing the space bar, which triggered the presentation of a scene image lasting for 3 s. The entire experiment lasted approximately 90 min. Note, in connection with an additional attention experiment, we conducted another eye-tracking experiment (see section ‘Attention experiment’ for more details).

### Measured variables and preprocessing

In line with de Haas et al.^5^ and manufacturer’s recommendations, we excluded fixations with onset latencies or durations shorter than 100 ms, as well as fixations that could not be assigned a label. This allowed us to restrict our analysis to object-directed fixations occurring after stimulus onset. To account for foveal extent and eye-tracking accuracy, a fixation was assigned to a labelled object pixel mask for which the distance between the fixation coordinate and pixel mask was within a tolerance margin of ∼0.5 dva (cf. ^5^). We then quantified the cumulative fixation duration (that is, dwell time) for each observer that fell on text elements or faces as the percentage of cumulative dwell time on any labelled pixel masks. Similarly, we quantified the percentage of labelled first fixations after image onset that fell on text elements or faces (cf. ^5^).

### Magnetic resonance imaging

We collected neuroimaging data at the Benders Institute of Neuroimaging at Justus-Liebig University using a 3-Tesla Siemens Prisma scanner and a 64-channel head coil.

#### Structural imaging

High-resolution anatomical images were obtained using a T1-weighted magnetization-prepared rapid acquisition gradient-echo sequence with a field of view (FoV) of 240 mm × 240 mm, an echo time (TE) of 3.53 ms and a repetition time (TR) of 1,880 ms. Further parameters for obtaining functional data were as follows: inversion time of 949 ms, in-plane resolution of 0.94 mm × 0.94 mm, number of slices 176, slice thickness of 0.94 mm and flip angle (FA) of 8°. Magnetic field perturbations were accounted for by measuring a field map with the following scan parameters: FoV of 220 mm × 220 mm, TE (1) of 10 ms, TE (2) of 12.46 ms, TR of 1,000 ms, in-plane resolution of 2 mm × 2 mm, slice thickness of 3 mm, number of slices 40 (transversal) and FA of 90°.

#### Functional imaging

The functional data was acquired with the same scanner and head coil as the structural images. Functional images covered the whole brain and were obtained using a multiband echo-planar imaging sequence with the following scan parameters: TE of 30 ms; TR of 1,500 ms; FoV of 220 mm × 220 mm, in-plane resolution of 2.2 mm × 2.2 mm, 46 slices (descending) with a thickness of 2.2 mm and a distance factor of 20%, FA of 71°, in-plane acceleration factor of 2 and slice-acceleration factor of 2. We acquired 1,086 volumes of functional data per participant (181 per run).

We used a magnetic resonance imaging-compatible eye camera (12M-I, Arrington Research) to observe participants’ compliance with task requirements online (for example, to hold fixation and to not move the eyes). Stimuli were projected onto a screen in the scanner bore, just behind the head coil using an Epson EB-G5600 projector with a resolution of 1,024 pixels × 768 pixels and a refresh rate of 60 Hz.

#### Localizer experiment

During functional imaging, participants completed a total of six runs of a standard localizer experiment, in which stimuli of six different categories were displayed from the fLoc functional localizer package (faces, bodies, pseudowords, houses, cars and limbs; see ref. ^30^). Category stimuli were placed on a phase-scrambled background, which was generated from randomly selected images^30^. All stimuli were presented in greyscale, and the total image size including the background spanned 28 dva^2^. Individual images were presented only once in a trial but could be repeated randomly across trials. Images of one category were presented in 6-s blocks and were presented at category-specific rates (faces, 3.3 Hz; bodies, 4.5 Hz; pseudowords, 2 Hz; houses, 2.6 Hz; cars, 2 Hz; limbs, 2 Hz). This is following previous research, suggesting category-specific temporal processing and encoding differences^30^. At the beginning and end of each run, a baseline block was added, showing a grey luminance screen. Each run consisted of six blocks of each category. These blocks were shown in random order and intermixed with rest blocks showing only a grey background. Category blocks contained 12–27 stimuli, whereas rest blocks contained no stimuli. Participants were instructed to view the images while fixating on a central dot throughout. In each category block, one target was randomly interspersed, consisting of only the phase-scrambled background (that is, no object overlayed). The oddball task required participants to press a button within 1 s of the appearance of this target. Responses from two participants were excluded due to a button-box malfunction. For the remaining 59 participants, performance on the oddball task during scanning was high for both face (hit rate of 80%, s.d. of 15%) and word blocks (hit rate of 83%, s.d. of 13%). In a supplementary analysis, we found no correlations between performance in the oddball tasks and category-specific gaze tendencies or category distinctiveness (Supplementary Table 2).

### Assessing perceptual performance

A subset of our participants completed one or both of the following behavioural tasks: the CFMT in the long format^37^ and the German speed-reading task SLRT-2^38^.

#### Assessing face recognition ability

We assessed participants’ face recognition ability using the CFMT^37^, a self-paced recognition task consisting of trials that include separate learning and test phases. In each learning phase, participants study the identity of a previously unfamiliar face from three different viewpoints. In the test phase, a triplet of faces is shown, and participants are asked to identify the learned identity among distractor faces. The test becomes increasingly more difficult (unknown viewing angles, lighting and superimposed noise). The score is obtained by measuring the percentage of correct responses during the test phase.

#### Assessing reading ability

In a subset of native German speakers, we assessed their reading ability using the speed-reading test SLRT-2^38^. In this task, participants read one list of real words and one list of pseudowords as quickly and accurately as possible. The test has a time limit of one minute per list and scoring is based on the total number of (pseudo)words read minus the number of pronunciation errors.

### Attention experiment

A total of 60 naive participants (mean age 22.87 years, s.d. of 3.25 years; 8 male and 52 female) completed a shorter but otherwise similar version of the free-viewing experiment reported above (using 200 instead of 700 images; cf. Linka and de Haas^7^). Again, calibration was accepted only if the maximum error was ≤1.2 dva and the average error was ≤0.6 dva. The observed mean validation error was 0.35 dva (s.d. of 0.08 dva). These participants also completed a dual-task based on the localizer experiment. The task lasted ~10 min and contained 6 practice trials and 84 experimental trials total (2 × 42, alternating face and pseudowords). The experimental trials were divided into three equal blocks (28 trials each) with short breaks in between. The stimuli (spanning 28 dva^2^) and oddball task were identical to the face and pseudoword blocks in the localizer experiment, except for the addition of a secondary task. For the primary task, participants used the left arrow key to report the oddball target of a phase-scrambled background (that is, no object overlayed; same task as in the scanner). The new, secondary task (‘blob detector’) added a small (4.3 dva), mid-grey disk in one of the four image corners on one to three frames per trial. These secondary targets were reported using the right arrow key and considered a hit if they occurred within 1 s of onset. Per category, the blob counts followed a fixed distribution (3 trials with one blob occurence, 20 with two and 19 with three), yielding 200 secondary targets overall (100 during face trials and 100 during word trials). Image order and blob positions were identical for all participants (fixed assignment), and the minimum temporal spacing between any two targets (primary or secondary) was two primary stimuli (606 ms during face trials, 1,000 ms during word trials). Timing and keypresses were logged continuously. For each participant, we computed category-specific hit rates (for example, number of secondary targets in face blocks/hits) and the difference in hit rate between text and face blocks (text–face; that is, negative and positive values indicate an advantage during the face and text blocks, respectively). To assess split-half reliability, we computed this indicator and category-specific hit rates separately for the first and second halves of the experiment and correlated it across participants. Participants were compensated with course credit or payment (10 euros per hour). The study was conducted in accordance with the guidelines and regulations of the local ethics committee of Justus-Liebig-University Giessen (approval no. LEK-FB06), and all participants gave written informed consent before participation.

### Data analysis

We conducted all statistical analyses and generated all figures using MATLAB (R2024b, MathWorks) and SPM (https://www.fil.ion.ucl.ac.uk/spm/). For visualizations and definition of ROIs based on anatomical landmarks (cf. ^26^; Figs. 2 and 4), we processed each observer’s anatomical scans using FreeSurfer (http://surfer.nmr.mgh.harvard.edu/) to delineate the grey–white matter and grey matter pial boundaries. These boundaries were then used to reconstruct inflated cortical surfaces on which we projected statistical maps obtained using SPM.

### Definition of lateral and medial VTC regions

Following previous studies^18,26^, we defined lateral and medial VTC regions based on anatomical landmarks on the native inflated cortical surfaces of each participant’s hemisphere in FreeSurfer (http://surfer.nmr.mgh.harvard.edu/). The posterior boundary of both VTC ROIs was set along the middle section of the posterior transverse collateral sulcus, whereas the anterior boundary was aligned with the anterior tip of the mid-fusiform sulcus. However, we positioned this boundary slightly more anteriorly, as the face-selective region called mFus-faces is predominantly located in this region^18^. The lateral border of the lateral VTC ROI was placed along the lateral edge of the inferior temporal gyrus, whereas the medial and lateral VTC ROIs were separated by a boundary along the mid-fusiform sulcus. The medial border of the medial VTC ROI was set along the medial edge of the collateral sulcus. In line with reported effects in a previous study^26^, we focused our analysis related to category distinctiveness on the lateral VTC.

### Preprocessing of fMRI data

Functional data preprocessing was performed using SPM (https://www.fil.ion.ucl.ac.uk/spm/). Functional images were realigned and unwarped using voxel displacement maps derived from the field maps. Functional data were then co-registered to each participant’s structural scan and spatially smoothed with a 5-mm Gaussian kernel. Supplementary analyses confirmed that light smoothing led to more robust estimates of category-specific patterns (see also section ‘Reliability of multivariate patterns’ of the Supplementary Informationfor more details).

### Extraction of neural category selectivity

We specified a general linear model with boxcar regressors for each stimulus category and six nuisance regressors for estimated rigid motion parameters based on the realignment. Category regressors were convolved with the canonical haemodynamic response function as implemented in SPM. This allowed us to estimate the response amplitude for each of the six conditions at the voxel level. Specifically, we computed voxel-wise *t*-statistics for the contrast between a given category and the average of all others.

#### Analysis of category distinctiveness

For each category, multivoxel patterns were defined as vectors of the corresponding *t*-values across all voxels within a given ROI (lateral VTC in the left or right hemisphere). For each participant, we computed one representational similarity matrix (RSM^76^) per hemisphere by correlating multivoxel patterns from one half of the localizer runs (odd runs 1, 3 and 5) with those from the other half (even runs 2, 4 and 6). This yielded a 6 × 6 RSM for each hemisphere, with within-category correlations represented on the diagonal and between-category correlations on the off-diagonal (Fig. 2d). These matrices were then used to calculate face and word distinctiveness. These two categories were selected on the basis of consistent findings from prior research demonstrating robust and substantial individual differences in adults’ gaze tendencies for faces and words^5,7^ as well as the protracted development and behavioural relevance of corresponding representational distinctiveness^26^. The distinctiveness of a category is calculated as the within-category minus the average between-category similarity of multivariate responses with the other five categories, and the resulting values can theoretically range from −2 to 2.

#### Definition of fROIs and their individual size

We defined fROIs using the automated group-constrained subject-specific approach (cf. ^39,77,78^). This method provides unbiased, participant-specific fROIs and is highly suitable for our sample size (*N* = 61). First, individual first-level general linear models were estimated in MNI (Montreal Neurological Institute) space using unsmoothed data. We then used the spm_ss toolbox (https://www.nitrc.org/projects/spm_ss) (ref. ^78^) to generate binary activation maps for the contrasts of faces > other categories and words > other categories, respectively, both thresholded at *P* < 0.0001 (uncorrected). These binary maps were overlaid across participants, spatially smoothed (full width at half maximum (FWHM) of 6 mm) and thresholded to retain only voxels with at least 10% overlap across the sample. Resulting group-level parcels were restricted to those within which at least 60% (36/61) of participants showed significant activation. We compared resulting group-level peaks of respective parcels to previous literature and focused on the rFFA (MNI peak coordinates, +40, −47, −21; cf. ^39^) and lVWFA (MNI peak coordinates, −44, −66, −13; cf. ^60^; see Fig. 4a, for an illustration of fROI locations).

To quantify participant-specific activation, we performed a masked voxel-count analysis. Each participant’s *t*-contrast map (unsmoothed data; faces > other categories or words > other categories) was masked by the respective group-level parcel (rFFA or lVWFA). Within these constraints, we calculated the total number of significant voxels exceeding a threshold of *P* < 0.0001 (uncorrected; Fig. 4a,b).

### Statistics

We used Pearson correlations to test the split-half reliability (cf. ^5^) of individual gaze tendencies (proportions of first fixations and dwell time on faces or text elements) and rFFA or lVWFA sizes. As two metrics were used to quantify gaze tendencies, significance thresholds were Bonferroni-adjusted: we reported original *P* values throughout, but only values below *α* = 0.025 (0.05/2) are considered significant. Furthermore, we examined the relationships between-category distinctiveness, fROI sizes, gaze tendencies and behavioural performance on the CFMT and speed-reading tests.

To assess the respective significance of the observed correlations, we first computed the correlation coefficient between the two original vectors. Next, we generated a null distribution by randomly shuffling one of the vectors and recalculating the correlation coefficient with the unshuffled vector. This procedure was repeated 10,000 times to create a distribution of correlation coefficients expected under the null hypothesis of no association. Finally, we calculated the proportion of these randomly generated correlations that were as extreme or more extreme than the observed correlation, providing an empirical estimate of the *P* value. By default, reported *P* values are two-tailed unless otherwise specified in the text.

We tested two primary a priori hypotheses: first, individual face salience is linked to face distinctiveness in the right lateral VTC and second, individual text salience is linked to word distinctiveness in the left lateral VTC. We quantified category-specific individual salience as the individual proportions of first fixations and dwell time spent on corresponding stimuli during the free-viewing experiment. To maintain statistical rigour, we adjusted significance thresholds for the use of these two metrics using the method of Bonferroni. We report original *P* values but only interpret values below *α* = 0.025 (0.05/2) as significant. Note, we signalled significant results with asterisks in Supplementary Table 1. We also adopted a conservative stance by using two-sided tests, despite directional predictions for positive associations. We also tested the hypotheses that individual perceptual performance for reading and face recognition are linked to respective individual fixation tendencies and to corresponding representational distinctiveness. Here, we also corrected for the two salience measures tested (where applicable) but used one-sided tests. This decision was based on previous findings supporting the directional hypotheses (cf. ^14,15,26^) and the lower sample sizes for perceptual performance tests. Significance levels for associations with speed-reading scores were additionally corrected for the two reading measures used (pseudowords and words).

Complementing our main analyses, we explored associations across hemispheres and categories (for example face distinctiveness in left lateral VTC versus face salience and face distinctiveness in right lateral VTC versus text salience). These served as specificity controls to test if the observed relationships between neural and gaze patterns are hemisphere- and category-specific. Following reviewer suggestions, we also explored associations with lVWFA and rFFA sizes. For all these exploratory analyses, we applied the same corrections as for the main analysis to account for the two metrics of salience (proportion of first fixations and dwell time).

For the attention experiment, we additionally computed Bayesian Pearson correlations in JASP (Version 0.96; JASP Team^79^) using a Jeffreys–Zellner–Siow prior. The Bayes factors (BF_10_) were interpreted following Wetzels and Wagenmakers^80^, where values below 1 indicate evidence in favour of the null hypothesis.

### Sample size

Our sample size aimed for good sensitivity (power >0.85) for effect sizes of *r* ≥ 0.4. This was based on previously observed effect sizes for the relation between category-specific distinctiveness and perceptual skills among children^26^. Under our hypothesis, we expected somewhat larger effect sizes for the relationship between representational distinctiveness and gaze tendencies among adults, given that our estimates of distinctiveness were based on three times the number of localizer runs per data point (six instead of two; Nordt et al.^26^), the generally better signal-to-noise ratio of BOLD responses in adults (for example, Thomason et al.^81^) and the excellent internal consistency of individual differences in face- and word-fixation tendencies among adults^5,82^.

### Reporting summary

Further information on research design is available in the Nature Portfolio Reporting Summary linked to this article.

## Supplementary information

Supplementary Information Supplementary Methods, Results, Tables 1–3 and Figs. 1 and 2.

Reporting summary

Peer Review File

## Data Availability

The anonymized data used to generate the results in this study and generate Figs. 1–5 are freely available via OSF at https://osf.io/9fhxk/. The raw fMRI data generated in this study are protected by privacy laws and not available. However, raw or processed data may be shared with accredited researchers upon request.

## Code availability

MATLAB code and stimuli used to generate the figures and results are freely available via OSF at https://osf.io/9fhxk/.
