# Supplementary material for: Active vision is linked to category selectivity in the individual brain
Source: Nat Hum Behav. 2026 Jul 7;10(9):1808–20. doi: 10.1038/s41562-026-02494-5 (PMC13590408; doi:10.1038/s41562-026-02494-5)
Supplement: Supplementary file 1 [file 41562_2026_2494_MOESM1_ESM.pdf]

nature human behaviour

Supplementary information

https://doi.org/10.1038/s41562-026-02494-5

Active vision is linked to category selectivity in the individual brain

In the format provided by the authors and unedited

Contents

Supplementary Materials 2
Calculation of fMRI laterality index of category-specific responses 2
Exploratory searchlight analysis 2
Supplementary Results 4
Detailed results reported in the main text 4
Reliability of multivariate patterns 8
No observed correlation between oddball task accuracy and gaze tendencies or category distinctiveness 8
No robust correlations between lateralization of category-specific responses and gaze tendencies or behavioural performance 9
Whole brain searchlight analysis of category distinctiveness and its relation to gaze tendencies 10
References 14

Supplementary Materials

Calculation of fMRI laterality index of category-specific responses

To test a potential relationship between fixation tendencies and the lateralization of category-specific responses, we followed the approach described in Blauch et al. (2025)[1]. To identify visually responsive cortex, we first defined a stimulus-responsive mask. For each participant, we specified a first-level general linear model (GLM) contrast comparing the average of all six stimulus conditions (faces, bodies, words, houses, cars, limbs) to the blank condition (all stimuli > blank). The resulting t-map was thresholded at $p < .001$ (uncorrected, voxel-wise; extent threshold = 0 voxels) and binarized. The subsequent laterality analysis was restricted to the intersection of this stimulus-responsive mask and the anatomically defined ROIs: lateral and medial ventral temporal cortex (VTC) in the left and right hemispheres (cf. methods section 'Definition of lateral and medial VTC regions').

For each participant and each category-selective contrast (faces and words), we extracted the corresponding first-level t-map. Within the left and right stimulus-responsive VTC masks, we then computed the sum of positive t-values. This measure captures the overall category-selective response profile within the stimulus-responsive anatomical ROI, combining effect magnitude and spatial extent.

Hemispheric laterality was quantified with a laterality index (LI) defined as the relative difference of summed t-values between the left and right hemisphere. LI > 0 indicates left-lateralized activation for a given category, LI < 0 indicates right-lateralized activation, and LI ~ 0 indicates bilateral or weakly lateralized activation.

Exploratory searchlight analysis

For each participant, we computed whole-brain searchlight maps of neural distinctiveness separately for faces and words in native space. To this end, a spherical searchlight with a radius of 4 voxels (~8mm) was iteratively centred on each voxel of a given individual's grey-

matter-constrained image (SPM C1 image, thresholded at >.02). For each iteration, we computed category distinctiveness of responses intersecting with the sphere as described in Methods section ‘Analysis of category distinctiveness’. We then projected the resulting distinctiveness values onto the corresponding centre voxel. This analysis was implemented using the CoSMoMVPA toolbox[2].

The resulting distinctiveness maps for word and face-representations were normalized to MNI space using the participant-specific forward deformation fields estimated from each participant’s T1-weighted anatomical image in SPM 12 (Wellcome Trust Centre for Neuroimaging, London, UK) and restricted to cerebral cortex using the cortical mask of the Harvard-Oxford atlas (HOA; [3,4]).

At the group level, we first tested where category distinctiveness was reliably greater than zero across participants. For each stimulus category (words, faces), we entered the corresponding individual distinctiveness maps into a second-level one-sample t-test in SPM (random-effects GLM). This yielded whole brain statistical parametric maps identifying voxels in which mean distinctiveness values were significantly positive across participants. The resulting maps were thresholded with cluster-level FWE correction at $p < .05$, and significant clusters were saved as binary masks for subsequent analyses (word- and face-selective distinctiveness clusters, respectively).

We then tested whether inter-individual differences of representational distinctiveness within these clusters related to gaze tendencies. For this, we fitted second-level multiple regression models in SPM with the respective distinctiveness map as dependent images and the participant-specific gaze tendencies (first fixation or dwell time) as the covariate of interest. To restrict the analysis to regions that showed reliable category distinctiveness, we used the significant clusters from the corresponding group-sample t-test as an explicit mask in the regression model. We then computed t-contrasts testing for positive associations between category distinctiveness and gaze tendencies (first fixation and dwell time). Anatomical

regions associated with significant peak-level MNI coordinates were identified based on the Harvard-Oxford atlas (HOA; [3,4])

For visualizations, the group-level statistical t-maps were projected onto the Freesurfer fsaverage inflated cortical surface. This yielded a topography of cortical regions where local neural category distinctiveness covaried with individual differences in gaze tendencies.

Supplementary Results

Detailed results reported in the main text

Table S1

Overview of Results Reported in the Main Text

**Test**	**Result**	**95% confidence interval**	**Two-tailed**	**Alpha**
**Result section: Representational distinctiveness is linked to individual differences in gaze**				
Max-min ratio of face distinctiveness in the right lateral VTC	1.38			
Max-min ratio of word distinctiveness in the left lateral VTC	1.52			
Correlation between face distinctiveness in right lateral VTC and (1) first fixations and (2) dwell time on faces	(1) $r(59) = .38$, $p = .003$* (2) $r(59) = .30$, $p = .017$*	(1) [0.1452, 0.5630] (2) [0.1251, 0.4653]	yes	.025
Correlation between word distinctiveness in left lateral VTC and (1) first fixations and (2) dwell time on text	(1) $r(59) = .44$, $p < .001$* (2) $r(59) = .29$, $p = .023$*	(1) [0.2476, 0.6008] (2) [0.0824, 0.4853]	yes	.025
Correlation between word distinctiveness in right lateral VTC and (1) first fixations	(1) $r(59) = .35$, $p = .006$* (2) $r(59) = .14$, $p = .299$	(1) [0.1303, 0.5467] (2) [-0.0450, 0.3174]	yes	.025

and (2) dwell time on text				
Correlation between face distinctiveness in left lateral VTC and (1) first fixations and (2) dwell time on faces	(1) *r*(59) = .20, *p* = .124<br>(2) *r*(59) = .05, *p* = .726	(1) [-0.0376, 0.4242]<br>(2) [-0.2003, 0.2902]	yes	.025
Cross-correlations between word distinctiveness in left lateral VTC and (1) first fixations and (2) dwell time on faces	(1) *r*(59) = .16, *p* = .222<br>(2) *r*(59) = -.03, *p* = .818	(1) [-0.0787, 0.3985]<br>(2) [-0.2813, 0.2134]	yes	.025
Cross-correlations between face distinctiveness in right lateral VTC and (1) first fixations and (2) dwell time on text	(1) *r*(59) = -.36, *p* = .005*<br>(2) *r*(59) = -.24, *p* = .058	(1) [-0.5503, -0.1372]<br>(2) [-0.4271, -0.0379]	yes	.025
**Result section: Representational distinctiveness is linked to behavioural performance**				
correlation between face distinctiveness in right lateral VTC and the CFMT score	*r*(32) = .44, one-tailed *p* = .004*	[0.2044, 0.6427]	no	.05
correlation between word distinctiveness in left lateral VTC and reading-speed scores for (1) pseudowords and (2) words	(1) *r*(23) = .44, one-tailed p = .012*<br>(2) *r*(23) = .17, one-tailed *p* = .204	(1) [0.0311, 0.7218]<br>(2) [-0.2953, 0.6170]	no	.025
**Result section: The individual size of lVWFA and rFFA is linked to fixation tendencies and behavioural performance**				
Correlation between rFFA cluster size and (1) first fixations and (2) dwell time on faces	(1) *r*(59) = .39, *p* = .002*<br>(2) *r*(59) = .28, *p* = .031	(1) [0.1650, 0.5831]<br>(2) [0.0175, 0.5230]	yes	.025
Cross-correlation between rFFA cluster size and (1) first fixations and (2) dwell time on text	(1) *r*(59) = -.37, *p* = .004*<br>(2) *r*(59) = -.34, *p* = .007*	(1) [-0.5636, -0.1262]<br>(2) [-0.5243, -0.1321]	yes	.025

Correlation between rFFA cluster size and face distinctiveness in right lateral VTC	*r*(59) = .55, *p* < .001*	[0.3634, 0.6963]	yes	.05
Correlation between rFFA cluster size and CFMT score	*r*(32) = .37, one-tailed *p* = .014*	[0.0923, 0.5983]	no	.05
Correlation between lVWFA cluster size and (1) first fixations and (2) dwell time on text	(1) *r*(59) = .35, *p* = .006*<br>(2) *r*(59) = .26, *p* = .039	(1) [0.1453, 0.5411]<br>(2) [0.0642, 0.4542]	yes	.025
Cross-correlation between lVWFA cluster size and (1) first fixations and (2) dwell time on faces	(1) *r*(59) = .09, *p* = .514<br>(2) *r*(59) = .01, *p* = .962	(1) [-0.1425, 0.3056]<br>(2) [-0.2147, 0.2132]	yes	.025
Correlation between lVWFA cluster size and word distinctiveness in left lateral VTC	*r*(59) = .75, *p* < .001*	[0.6319, 0.8331]	yes	.05
Correlation between lVWFA cluster size and reading-speed scores for (1) words and (2) pseudowords	(1) *r*(23) = .10, one-tailed *p* = .319<br>(2) *r*(23) = .32, one-tailed *p* = .060	(1) [-0.3027, 0.5117]<br>(2) [-0.0507, 0.5939]	yes	.025
**Result section: Face salience is linked to face recognition performance**				
Max-min ratio of CFMT performance scores	*subsample*: 1.98;<br>*M* = 64.08, *SD* = 11.87<br>*extended sample:* 2.33;<br>*M* = 64.37, *SD* = 11.95)			
correlation between CFMT scores and (1) proportion of first fixations and (2) proportion of dwell time on faces	(1) *subsample*: *r*(32) = .23, one-tailed *p* = .094<br>*extended sample:* *r*(67) = .39, one-tailed *p* < .001*<br>*(2) subsample:* *r*(32) = *.16, one-tailed p = .174*<br>*extended sample:* *r*(67) = .18, one-tailed *p* = .077	(1) *subsample:* [-0.0662, 0.4925]<br>*extended sample:* [0.1679, 0.5767]<br><br>(2) *subsample:* [-0.1726, 0.4917]<br>*extended sample:* [-0.0438, 0.3849]	No	.025

Max-min ratio of speed-reading test scores for (1) words and (2) pseudowords	(1) *subsample*: 1.97; *M* = 118.40, *SD* = 16.17<br>*extended sample:* 2.11; *M* = 111.35, *SD* = 18.07<br>(2) *subsample*: 2.15; *M* = 77.92, *SD* = 14.57<br><br>*extended sample*: 3.52; *M* = 74.68, *SD* = 16.36			
correlation between speed-reading test scores for (a) words and (b) pseudowords and (1) first fixations and (2) proportion of dwell time on text	(1a) *subsample*: r(23) = .50, one-tailed p = .004*<br>*extended sample:* *r*(63) = .-04, one-tailed *p* = .386<br>(1b) *subsample:* *r*(23) = .69, one-tailed *p* <.001*<br>*extended sample:* *r*(63) = .19, one-tailed *p* = .065<br><br>2a) *subsample*: r(23) = .34, one-tailed p = .042<br>*extended sample:* *r*(63) = .-17, one-tailed *p* = .090<br>(2b) *subsample:* *r*(23) = .39, one-tailed *p* =.025<br>*extended sample:* *r*(63) = .04, one-tailed *p* = .383	*(1a) subsample:* [0.2336, 0.7410]<br><br>*extended sample:* [-0.3098, 0.2390]<br><br>*(1b) subsample:* [0.4264, 0.8396]<br><br>*extended sample:* [-0.0448, 0.4249]<br><br>*(2a) subsample:* [-0.0714, 0.6644]<br><br>*extended sample:* [-0.4428, 0.1139]<br><br>*(2b) subsample:* [-0.1370, 0.6730]<br><br>*extended sample:* [-0.2158, 0.2636]	No	.0125

Note. * indicates significant correlations. The significance level was Bonferroni-corrected to account for the use of two salience metrics (proportion of first fixations and dwell time), and two speed reading scores (words and pseudowords). Adjusted significance levels and test directionality are denoted for each comparison.

Reliability of multivariate patterns

We compared the reliability of category-specific multivariate patterns in the individual brain before and after smoothing with a kernel of 5 mm. Averaged across participants, mean pattern reliability in *right* lateral VTC *without smoothing* was .83 (*SD* = .08) for faces, .71 (*SD* = .12) for bodies, .69 (*SD* = .11) for words, .67 (*SD* = .15) for houses, .45 (*SD* = .17) for cars, and .64 (*SD* = .14) for limbs.

With smoothing, mean category reliability in *right* lateral VTC was .94 (*SD* = .04) for faces, .87 (*SD* = .08) for bodies, .86 (*SD* = .09) for words, .86 (*SD* = .09) for houses, .67 (*SD* = .19) for cars, and .84 (*SD* = .10) for limbs.

Without smoothing, mean category reliability in *left* lateral VTC was .81 (*SD* = .08) for faces, .64 (*SD* = .14) for bodies, .78 (*SD* = .10) for words, .66 (*SD* = .16) for houses, .48 (*SD* = .15) for cars, and .67 (*SD* = .14) for limbs.

With smoothing, mean category reliability in *left* lateral VTC was .92 (*SD* = .05) for faces, .82 (*SD* = .10) for bodies, .90 (*SD* = .05) for words, .86 (*SD* = .09) for houses, .68 (*SD* = .13) for cars, and .85 (*SD* = .12) for limbs.

No observed correlation between oddball task accuracy and gaze tendencies or category distinctiveness

Here we tested whether performance in the oddball task during category-specific blocks of the fMRI localiser session correlated with corresponding gaze tendencies or category distinctiveness. This was not the case (see Table S2). Note, two participants had to be excluded from this analysis due to button malfunction during the fMRI localizer experiment.

Table S2

Correlations between Oddball Task Accuracy, Gaze Tendencies and Category Distinctiveness

Correlation	Result
correlation between Oddball accuracy in face blocks and (1) proportion of first	(1) $r(56) = .14$, $p = .287$<br>(2) $r(56) = .07$, $p = .624$

fixations and (2) proportion of dwell time on faces	
correlation between Oddball accuracy in word blocks and (1) proportion of first fixations and (2) proportion of dwell time on text	(1) $r(56) = .01$, $p = .945$<br>(2) $r(56) = -.03$, $p = .808$
correlation between Oddball accuracy in face blocks and face distinctiveness in the right lateral VTC	$r(56) = .05$, $p = .728$
correlation between Oddball accuracy in word blocks and word distinctiveness in the left lateral VTC	$r(56) = .15$, $p = .264$

No robust correlations between lateralization of category-specific responses and gaze tendencies or behavioural performance

We explored hemispheric laterality indices for face- and word- selective responses in VTC (cf. [1]) and tested whether individual differences in laterality were associated with gaze tendencies and behavioural performance. As expected, face-selective activation was on average right lateralized (mean LI_faces = -0.15, SD = 0.28; negative values indicate right hemisphere dominance; $t(60) = -4.14$, $p < .001$), whereas word-selective activation was left-lateralized (mean LI_words = 0.45, SD = 0.26; $t(60) = 13.34$, $p < .001$). Importantly, participants varied substantially in the degree of category laterality.

To illustrate this variability, Figure 2 highlights two participants with opposite gaze tendencies. For face selectivity, participant 40 (who showed a stronger tendency to look at faces and a relatively high CFMT score) exhibited a stronger right hemispheric bias (LI_faces = -0.10) relative to participant 4 (who showed a stronger tendency to look at text and relatively high pseudoword reading score; LI_faces = -.02).

However, across participants, we found no robust evidence for relationships between laterality indices and gaze tendencies or behavioural performance (see Table S3). The only (negative) association was between LI_words and the proportion of first fixations on text, $r(58) = -.26$, two-tailed $p = .041$, but this effect did not survive correction for multiple comparisons (Bonferroni-corrected significance threshold $\alpha = .025$).

Furthermore, we found no meaningful association between LI_faces and LI_words ($r(58) = -.10$, $p = .433$), nor between word distinctiveness in left lateral VTC and face distinctiveness in right lateral VTC ($r(58) = -.04$, $p = .768$). Together, these results align with prior work reporting no evidence for local face–word competition in VTC[1].

Table S3

Correlations between Category Lateralization Index and Gaze Tendencies and Behavioural Performance

**Correlation**	**Result**
correlation between LI_faces and (1) proportion of first fixations and (2) proportion of dwell time on faces	(1) $r(58) = -.15$, two-tailed $p = .233$<br>(2) $r(58) = -.20$, two-tailed $p = .115$
correlation between LI_words and (1) proportion of first fixations and (2) proportion of dwell time on text	(1) $r(58) = -.26$, two-tailed $p = .041$<br>(2) $r(58) = -.12$, two-tailed $p = .353$
cross-correlation between LI_faces and (1) proportion of first fixations and (2) proportion of dwell time on text	(1) r(58) = .25, two-tailed $p = .055$<br>(2) $r(58) = .12$, two-tailed $p = .367$
cross-correlation between LI_words and (1) proportion of first fixations and (2) proportion of dwell time on faces	(1) $r(58) = .09$, two-tailed $p = .477$<br>(2) $r(58) = .20$, two-tailed $p = .132$
correlation between LI_faces and CFMT scores (N = 34)	$r(32) = -.10$, two-tailed $p = .588$
correlation between LI_words and reading test scores for pseudowords (N = 25)	$r(23) = -.20$, two-tailed $p = .329$
correlation between LI_words and reading test scores for words (N = 25)	$r(23) = -.14$, two-tailed $p = .508$

Whole brain searchlight analysis of category distinctiveness and its relation to gaze tendencies

Face distinctiveness was reliably greater than zero across participants in large parts of the inferior temporal cortex and parietal areas (Figures S1a). In the exploratory analysis relating face distinctiveness to the proportion of first fixations on faces, no significant clusters survived family-wise error (FWE) correction. For the correlation with dwell time on faces, a

cluster located in the right temporal occipital fusiform cortex (MNI peak coordinates xyz = 32, -48, -22; $t(59) = 3.98$; $p(\text{corrected}) = .007$; cluster size: k = 20; cf. Figure S1c) showed a correlation. The location of this peak is more medial than that we identified as rFFA using the automated Group-constrained Subject-Specific (GSS) approach, but corresponds to rFFA coordinates reported by others (cf. Spiridon et al., 2006[5]).

Figure S1: Searchlight for face distinctiveness and gaze tendencies

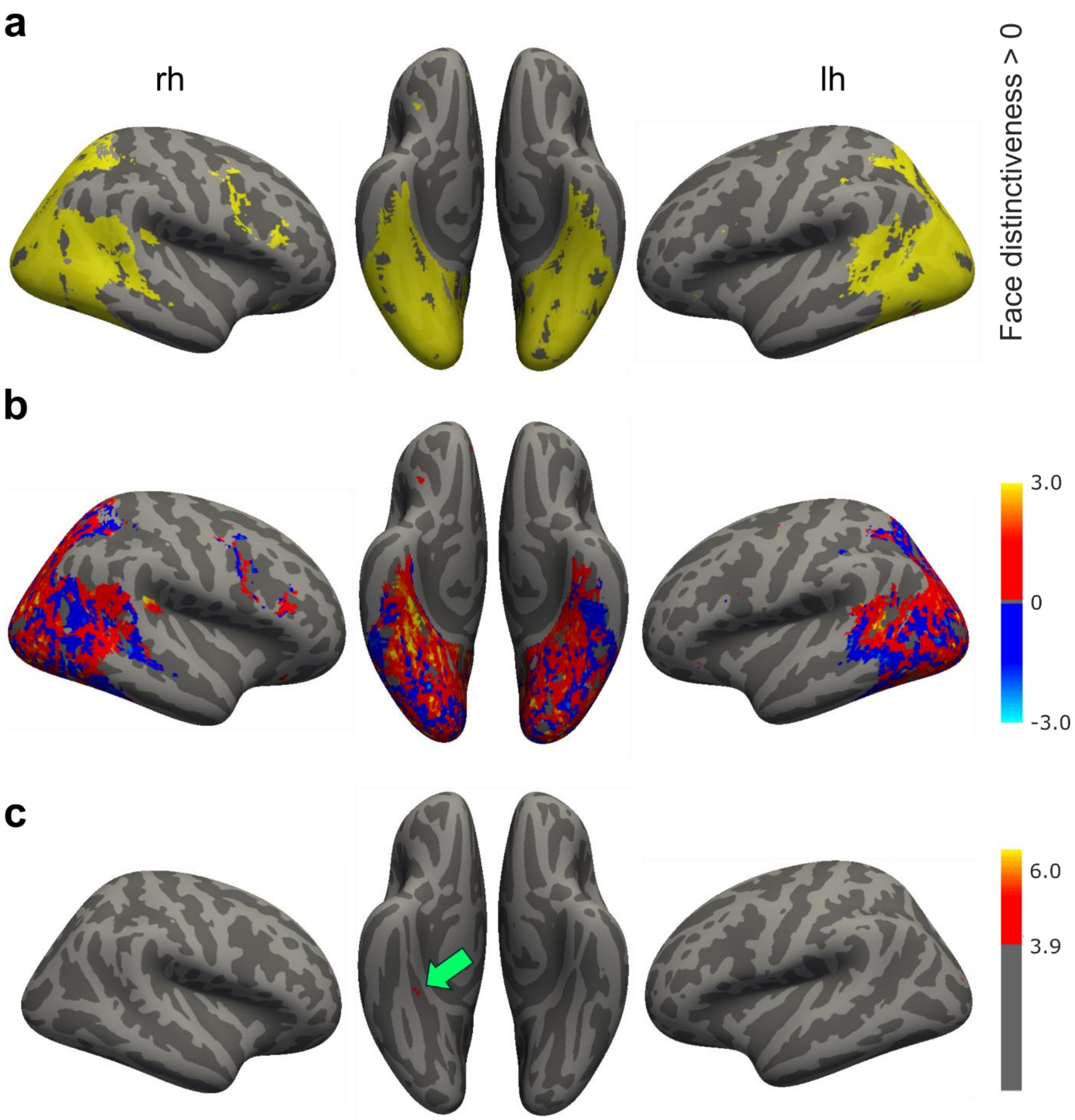

All panels show group-level patterns ($N = 61$). (a) Second-level GLM contrast testing face distinctiveness > 0 in the left (lh) and right (rh) hemispheres. The resulting binary map was used as a mask for the subsequent GLM analyses. (b, c) Overlays of t-contrast corresponding to the correlation between face distinctiveness and the proportion of dwell time on faces. The same contrast is shown both, without (b) and with (c) a minimum threshold applied, to give the complete picture and highlight statistically significant results (cf.

different inset colour bars on the right, showing corresponding t-values). The single significant cluster in rFFA that survived FWE corrections (*p*(corrected) = .007) is marked by a green arrow in (c); MNI peak coordinates: 32, -48, -22; temporal occipital fusiform cortex (TOF).

Word distinctiveness was reliably greater than zero across participants in large parts of the inferior temporal cortex, parietal and frontal areas (Figures S2a). For the relationship between word distinctiveness and the proportion of first fixations on text, we observed six significant clusters. The first temporooccipital cluster corresponded well to the left visual word from area (lVWFA; cf. [6]; MNI peak coordinates xyz = -50, -46, -28; $t(59) = 5.18$; $p(\text{corrected}) < .001$; cluster size: k = 34; cf. no. 1 in Figure S2c). A second significant cluster emerged in the left superior temporal gyrus (STGa; xyz = -62, -2, -4; $t(59) = 4.11$; $p(\text{corrected}) = .026$; cluster size: k = 53; cf. no. 2 in Figure S2c). Additionally, two significant clusters were identified in the left inferior frontal gyrus (IFGpt; xyz = -50, 30, 18; $t(59) = 3.95$; $p(\text{corrected}) = .002$; cluster size: k = 29; cf. no. 3 in Figure S2c, and IFGpo; xyz = -50, 8, 12; $t(59) = 3.62$; $p(\text{corrected}) = .011$; cluster size: k = 21; cf. no. 4 in Figure S2c). These regions are established components of the reading network[7,8]. A fifth and sixth significant cluster emerged in locations of the right temporal occipital fusiform cortex (TOF; xyz = 30, -46, -20; $t(59) = 3.74$; $p(\text{corrected}) = .032$; cluster size: k = 17; cf. no. 5 in Figure S2c) and lingual gyrus (xyz = 16, -86, -8; $t(59) = 4.50$; $p(\text{corrected}) = .007$; cluster size: k = 23; cf. no. 6 in Figure S2c). Previous reports found these regions to be active during letter and word processing as well[9]. No cluster survived FWE correction for the relationship between word distinctiveness and proportion of dwell time on text.

Figure S2: Searchlight for word distinctiveness and gaze tendencies

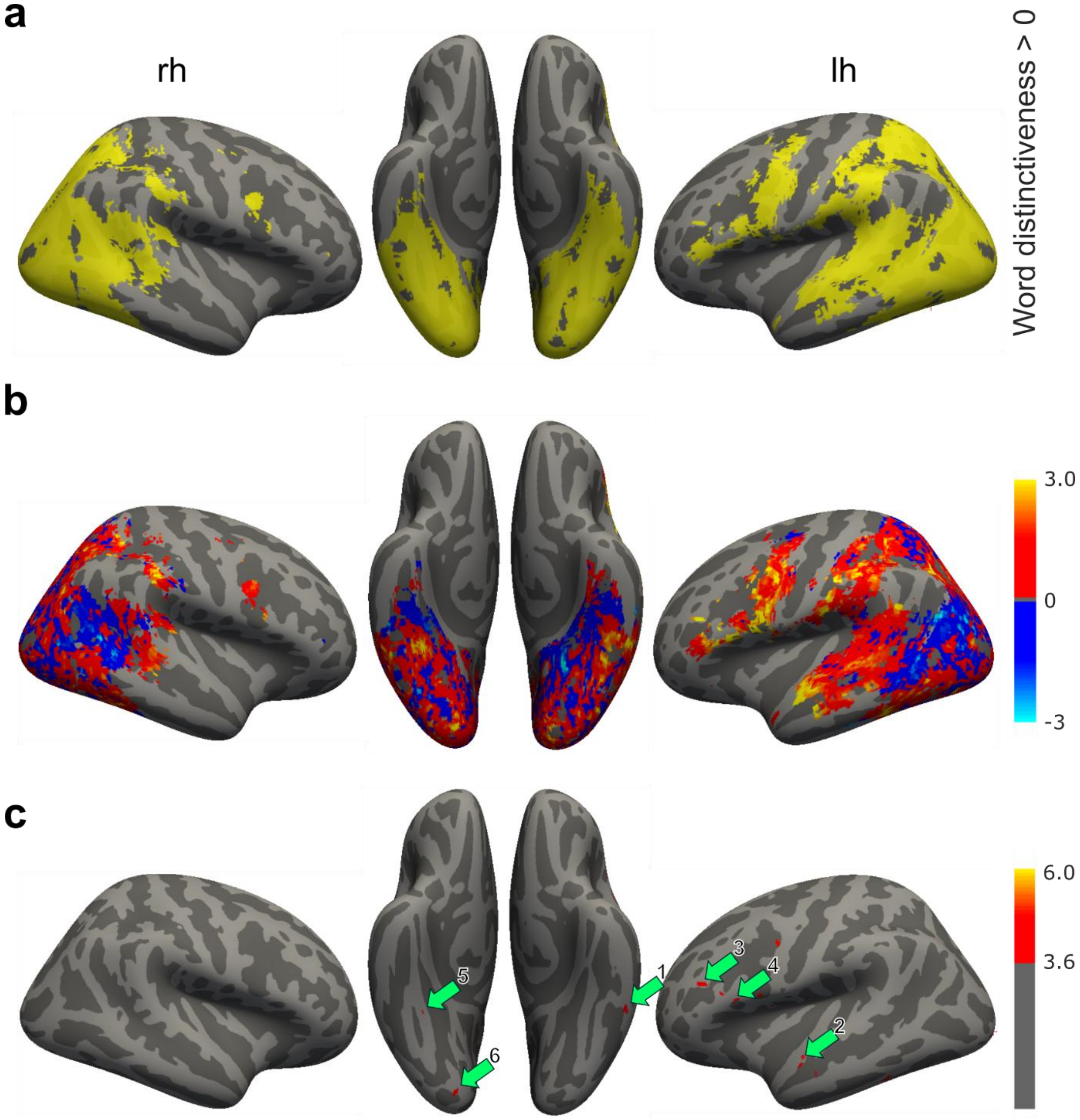

All panels show group-level patterns (N = 61). (a) Second-level GLM contrast testing word distinctiveness > 0 in the left (lh) and right (rh) hemispheres. The resulting binary map was used as a mask for the subsequent GLM analyses. (b, c) Overlays of t-contrasts showing the correlation between word distinctiveness and the proportion of first fixations on text. The same contrast is shown both, without (b) and with (c) a minimum threshold applied, to give the complete picture and highlight statistically significant results (cf. different inset colour bars on the right, showing corresponding t-values). The clusters in (c) that survived FWE corrections are marked by green arrows and corresponding numbers; MNI peak coordinates: 1) -50, -46, -28; inferior temporal gyrus, temporooccipital part (ITGtp), p(corrected) < .001; 2) -62, -2, -4; superior temporal gyrus, anterior division (STGa), p(corrected) = .026; 3) -50, 30, 18; inferior frontal gyrus, pars triangularis (IFGpt), p(corrected) = .002; 4) -50, 8, 12; inferior frontal gyrus, pars opercularis (IFGpo), p(corrected) = .011; 5) 30, -46, -20, temporal occipital fusiform cortex (TOF), p(corrected) = .032; 6) 16, -86, -8, occipital fusiform gyrus (OFG), p(corrected) = .007.

References

1. Blauch, N. M., Plaut, D. C., Vin, R. & Behrmann, M. Individual variation in the functional lateralization of human ventral temporal cortex: Local competition and long-range coupling. *Imaging Neurosci.* **3**, imag_a_00488 (2025).
2. Frontiers | CoSMoMVPA: Multi-Modal Multivariate Pattern Analysis of Neuroimaging Data in Matlab/GNU Octave. https://www.frontiersin.org/journals/neuroinformatics/articles/10.3389/fninf.2016.00027/full.
3. Desikan, R. S. *et al.* An automated labeling system for subdividing the human cerebral cortex on MRI scans into gyral based regions of interest. *NeuroImage* **31**, 968–980 (2006).
4. Jenkinson, M., Beckmann, C. F., Behrens, T. E. J., Woolrich, M. W. & Smith, S. M. FSL. *NeuroImage* **62**, 782–790 (2012).
5. Spiridon, M., Fischl, B. & Kanwisher, N. Location and spatial profile of category-specific regions in human extrastriate cortex. *Hum. Brain Mapp.* **27**, 77–89 (2006).
6. Dehaene, S. *et al.* How Learning to Read Changes the Cortical Networks for Vision and Language. *Science* **330**, 1359–1364 (2010).
7. Turker, S., Fumagalli, B., Kuhnke, P. & Hartwigsen, G. The ‘reading’ brain: Meta-analytic insight into functional activation during reading in adults. *Neurosci. Biobehav. Rev.* **173**, 106166 (2025).
8. Jobard, G., Crivello, F. & Tzourio-Mazoyer, N. Evaluation of the dual route theory of reading: a metanalysis of 35 neuroimaging studies. *NeuroImage* **20**, 693–712 (2003).
9. Protopapas, A. *et al.* Evaluating cognitive models of visual word recognition using fMRI: Effects of lexical and sublexical variables. *NeuroImage* **128**, 328–341 (2016).
