# Supplementary material for: Active vision is linked to category selectivity in the individual brain
Source: Nat Hum Behav. 2026 Jul 7;10(9):1808–20. doi: 10.1038/s41562-026-02494-5 (PMC13590408; doi:10.1038/s41562-026-02494-5)
Supplement: Supplementary file 2 [file 41562_2026_2494_MOESM2_ESM.pdf]

nature portfolio

Corresponding author(s): Diana Kollenda

Last updated by author(s): April 01, 2026

Reporting Summary

Nature Portfolio wishes to improve the reproducibility of the work that we publish. This form provides structure for consistency and transparency in reporting. For further information on Nature Portfolio policies, see our Editorial Policies and the Editorial Policy Checklist.

Statistics

For all statistical analyses, confirm that the following items are present in the figure legend, table legend, main text, or Methods section.

n/a	Confirmed	
☐	☒	The exact sample size (n) for each experimental group/condition, given as a discrete number and unit of measurement
☐	☒	A statement on whether measurements were taken from distinct samples or whether the same sample was measured repeatedly
☐	☒	The statistical test(s) used AND whether they are one- or two-sided<br>*Only common tests should be described solely by name; describe more complex techniques in the Methods section.*
☒	☐	A description of all covariates tested
☐	☒	A description of any assumptions or corrections, such as tests of normality and adjustment for multiple comparisons
☐	☒	A full description of the statistical parameters including central tendency (e.g. means) or other basic estimates (e.g. regression coefficient) AND variation (e.g. standard deviation) or associated estimates of uncertainty (e.g. confidence intervals)
☐	☒	For null hypothesis testing, the test statistic (e.g. F, t, r) with confidence intervals, effect sizes, degrees of freedom and P value noted<br>*Give P values as exact values whenever suitable.*
☒	☐	For Bayesian analysis, information on the choice of priors and Markov chain Monte Carlo settings
☒	☐	For hierarchical and complex designs, identification of the appropriate level for tests and full reporting of outcomes
☐	☒	Estimates of effect sizes (e.g. Cohen's d, Pearson's r), indicating how they were calculated

Our web collection on statistics for biologists contains articles on many of the points above.

Software and code

Policy information about availability of computer code

Data collection	The Cambridge Face Memory Test was completed using testable.org. The speed reading test was completed manually using paper and pen. Eye tracking and fMRI localiser data were collected using Psychtoolbox version 3.0.16 and MATLAB (R2021b, MathWorks, Natick, MA).
Data analysis	We conducted all statistical analyses and generated all figures using MATLAB (R2024b, MathWorks, Natick, MA) and SPM 12 (https://www.fil.ion.ucl.ac.uk/spm/). For visualizations and anatomical definitions of regions of interest, we processed each observer's anatomical scans using FreeSurfer v7.4.1 (http://surfer.nmr.mgh.harvard.edu/). We defined functional regions of interest (fROIs) using the automated Group-constrained Subject-Specific (GSS) approach (cf. Julian, et al., 2012; Fedorenko et al., 2010; Nieto-Castañón & Fedorenko, 2012). For the attention experiment, we additionally computed Bayesian Pearson correlations in JASP (Version 0.96; JASP Team, 2026). The exploratory searchlight analysis was implemented using the CoSMoMVPA toolbox. MATLAB code and stimuli used to generate the figures and results are freely available on OSF: https://osf.io/9fhxk/.

For manuscripts utilizing custom algorithms or software that are central to the research but not yet described in published literature, software must be made available to editors and reviewers. We strongly encourage code deposition in a community repository (e.g. GitHub). See the Nature Portfolio guidelines for submitting code & software for further information.

Data

Policy information about availability of data

All manuscripts must include a data availability statement. This statement should provide the following information, where applicable:

- Accession codes, unique identifiers, or web links for publicly available datasets
- A description of any restrictions on data availability
- For clinical datasets or third party data, please ensure that the statement adheres to our policy

The anonymized data used to generate the results in this study and generate the main figures are freely available from https://osf.io/9fhxk/. The raw (f)MRI data generated in this study are protected by privacy laws and not available. The data availability statement is provided in the manuscript.

Research involving human participants, their data, or biological material

Policy information about studies with human participants or human data. See also policy information about sex, gender (identity/presentation), and sexual orientation and race, ethnicity and racism.

Reporting on sex and gender	We recruited 102 healthy participants (30 male, 69 female, 3 diverse), with gender determined via self-report prior to the first eye-tracking block. All participants provided written informed consent for storage and sharing of their individual-level data. Although we aimed for gender balance, the final sample was not balanced. As gender was not part of our hypotheses or study design, no gender-based analyses were conducted. Moreover, the sample size was insufficient to draw meaningful conclusions about sex or gender effects.
Reporting on race, ethnicity, or other socially relevant groupings	We neither collected nor reported this data in our study.
Population characteristics	We recruited a total of 102 healthy participants with the following mean age = 24.64 years (SD = 4.58)
Recruitment	Participants were recruited via the Justus Liebig University Giessen mailing list, primarily targeting students and university employees.
Ethics oversight	The study was approved by the local ethics committee of the Justus-Liebig-University Giessen (LEK-FB06) and was conducted in accordance with the relevant guidelines and regulations.

Note that full information on the approval of the study protocol must also be provided in the manuscript.

Field-specific reporting

Please select the one below that is the best fit for your research. If you are not sure, read the appropriate sections before making your selection.

☒ Life sciences ☐ Behavioural & social sciences ☐ Ecological, evolutionary & environmental sciences

For a reference copy of the document with all sections, see nature.com/documents/nr-reporting-summary-flat.pdf

Life sciences study design

All studies must disclose on these points even when the disclosure is negative.

Sample size	Our sample size aimed for good sensitivity (power > .85) for effect sizes of r >= .4. This was based on previously observed effect sizes for the relation between category-specific distinctiveness and perceptual skills among children (Nordt et al., 2023, Nat Comms). Under our hypothesis, we expected somewhat larger effect sizes for the relationship between representational distinctiveness and gaze tendencies among adults, given our estimates of distinctiveness were based on three times the number of localizer runs per data point (six instead of two; Nordt et al, 2023); the generally better signal-to-noise ratio of BOLD responses in adults (e.g. Thomason et al., 2005, Neuroimage); and the excellent internal consistency of individual differences in face- and word-fixation tendencies among adults (de Haas et al, 2019, PNAS; de Haas, 2018 Front. Hum. Neurosci.).
Data exclusions	For the eye-tracking data, we excluded fixations with onset latencies or durations shorter than 100 ms, as well as fixations that could not be assigned a label. This is in line with de Haas et al. (2019) and manufacturer's recommendations, allowed us to restrict our analysis to object-directed fixations occurring after stimulus onset.
Replication	Previous studies using the same eye-tracking paradigm and stimuli have demonstrated excellent internal consistency of individual differences in face- and word-fixation tendencies among adults (de Haas et al., 2019, PNAS), as well as temporal stability (Linka & de Haas, 2020, JoV). In our neuroimaging experiment, we employed a standard localizer paradigm (Stigliani et al., 2015, J. Neurosci.) and replicated in an adult sample the findings of Nordt et al. (2023, Nat. Commun.), namely that word distinctiveness in the left and face distinctiveness in the right lateral VTC are linked to reading and face recognition performance. We also replicated the association between the tendency to first fixate on faces in scenes and face recognition performance (de Haas et al., 2019). In contrast, the relationship between the tendency to first fixate on text in scenes and reading performance reached significance only in the subsample of 25 participants who also took part in the fMRI session, but not in the extended sample of 65 participants. This renders the evidence for a link between gaze behavior and reading performance

inconclusive. Strong effects, however, appear unlikely given the high internal consistency of both gaze and performance measures (cf. Moll & Landerl, 2010). Nonetheless, as these experiments were partly limited by the availability of small behavioral subsamples, the findings should be interpreted with caution.

Randomization	Because our focus was on individual differences across the full sample, participants were not assigned to experimental groups.
Blinding	See above.

Reporting for specific materials, systems and methods

We require information from authors about some types of materials, experimental systems and methods used in many studies. Here, indicate whether each material, system or method listed is relevant to your study. If you are not sure if a list item applies to your research, read the appropriate section before selecting a response.

Materials & experimental systems

n/a	Involved in the study
☒	☐ Antibodies
☒	☐ Eukaryotic cell lines
☒	☐ Palaeontology and archaeology
☒	☐ Animals and other organisms
☒	☐ Clinical data
☒	☐ Dual use research of concern
☒	☐ Plants

Methods

n/a	Involved in the study
☒	☐ ChIP-seq
☒	☐ Flow cytometry
☐	☒ MRI-based neuroimaging

Plants

Seed stocks	*Report on the source of all seed stocks or other plant material used. If applicable, state the seed stock centre and catalogue number. If plant specimens were collected from the field, describe the collection location, date and sampling procedures.*
Novel plant genotypes	*Describe the methods by which all novel plant genotypes were produced. This includes those generated by transgenic approaches, gene editing, chemical/radiation-based mutagenesis and hybridization. For transgenic lines, describe the transformation method, the number of independent lines analyzed and the generation upon which experiments were performed. For gene-edited lines, describe the editor used, the endogenous sequence targeted for editing, the targeting guide RNA sequence (if applicable) and how the editor was applied.*
Authentication	*Describe any authentication procedures for each seed stock used or novel genotype generated. Describe any experiments used to assess the effect of a mutation and, where applicable, how potential secondary effects (e.g. second site T-DNA insertions, mosiacism, off-target gene editing) were examined.*

Magnetic resonance imaging

Experimental design

Design type	The neuroimaging study included six functional runs of a standard localizer experiment (Stigliani et al., 2015, J. Neurosci.), using a block design with 6-second trials in which participants viewed images from a single category.
Design specifications	Images of one category (faces, bodies, characters, houses, cars, limbs; cf. Stigliani et al., 2015, J. Neurosci.) were presented in 6 second blocks. Per run, six blocks of each category were shown in random order and in each category block, one target was randomly interspersed, consisting of only the phase-scrambled background (i.e. no object overlayed). At the beginning and end of each run, a baseline block was added, showing a grey luminance screen.
Behavioral performance measures	Participants were instructed to view the images while fixating on a central dot. The oddball task required participants to press a button whenever an image containing only the phase-scrambled background appeared.

Acquisition

Imaging type(s)	structural and functional
Field strength	3 Tesla
Sequence & imaging parameters	We collected neuroimaging data at the Benders Institute of Neuroimaging (BION) at Justus-Liebig University using a 3-Tesla Siemens Prisma scanner and a 64-channel head coil.<br>Structural imaging<br>High-resolution anatomical images were obtained using a T1-weighted magnetization-prepared rapid acquisition gradient-echo (MPRAGE) sequence with a Field of view (FoV) of 240 × 240 mm, an echo time (TE) of 3.53 ms and a repetition time (TR) of 1880 ms. Further parameters for obtaining functional data were as follows: inversion time = 949 ms, in-plane resolution = 0.94 mm × 0.94 mm, number of slices = 176, slice thickness = 0.94 mm, flip angle (FA) = 8°.

Magnetic field perturbations were accounted for by measuring a field map with the following scan parameters: FoV = 220 × 220 mm, TE (1) = 10 ms, TE (2) = 12.46 ms, TR = 1000 ms, in-plane resolution = 2 × 2 mm, slice thickness = 3 mm, number of slices = 40 (transversal), FA = 90°
Functional imaging
The functional data was acquired with the same scanner and head coil as the structural images. Functional images covered the whole brain and were obtained using a multiband echo-planar imaging sequence (EPI) with the following scan parameters: TE = 30 ms; TR = 1500 ms; FoV = 220 × 220 mm, in-plane resolution = 2.2 mm × 2.2 mm, 46 slices (descending) with a thickness of 2.2 mm and a distance factor of 20%, FA = 71°, in-plane acceleration factor = 2 and slice-acceleration factor = 2. We acquired 1086 volumes of functional data per participant (181 per run).

Area of acquisition: Structural and functional images covered the whole brain.

Diffusion MRI: ☐ Used ☒ Not used

Preprocessing

Preprocessing software: Functional data preprocessing was performed using SPM 12 (https://www.fil.ion.ucl.ac.uk/spm/). Functional images were realigned and unwarped using voxel displacement maps derived from the field maps. Functional data were then co-registered to each subject's structural scan and spatially smoothed with a 5 mm Gaussian kernel.

Normalization: Data normalization was applied to the functional regions of interest (fROI) and exploratory searchlight analyses; however, category distinctiveness was calculated using non-normalized data,

Normalization template: MNI

Noise and artifact removal: We specified a general linear model (GLM) with boxcar regressors for each stimulus category and six nuisance regressors for estimated rigid motion parameters based on the realignment.

Volume censoring: n/a

Statistical modeling & inference

Model type and settings: For each participant, we computed one representational similarity matrix (RSM (Kriegeskorte et al, 2008); per hemisphere by correlating MVPs from one half of the localizer runs (odd runs: 1, 3, and 5) with those from the other half (even runs: 2, 4, and 6). This yielded a 6 × 6 RSM for each hemisphere, with within-category correlations represented on the diagonal and between-category correlations on the off-diagonal. These matrices were then used to calculate face and word distinctiveness. The distinctiveness of a category is calculated as the within category minus the average between-category similarity of multivariate responses with the other five categories.

Effect(s) tested: We used Pearson correlations to test the split-half reliability (cf. de Haas et al., 2019, PNAS) of individual gaze behaviour (proportions of first fixations and dwell time on faces or text elements) as well as whether category distinctiveness is related to gaze behaviour, and behavioural performance on the CFMT and speed-reading test (cf. Nordt et al, 2023, Nat Comms).

Specify type of analysis: ☐ Whole brain ☒ ROI-based ☐ Both

Anatomical location(s): Following previous studies (Weiner & Grill-Spector, Psychological research, 2013; Nordt et al., 2023, Nat Comms), we defined lateral and medial VTC regions based on anatomical landmarks on inflated cortical surfaces of each participant’s hemisphere in FreeSurfer v7.4.1 (http://surfer.nmr.mgh.harvard.edu/). In line with reported effects in a previous study (Nordt et al., 2023), we focused our analysis on the lateral VTC in the right and left hemisphere.

Statistic type for inference (See Eklund et al. 2016): We specified a general linear model (GLM) with boxcar regressors for each stimulus category and six nuisance regressors for estimated rigid motion parameters based on the realignment. Category regressors were convolved with the canonical hemodynamic response function as implemented in SPM. This allowed us to estimate the response amplitude for each of the six conditions at the voxel level. Specifically, we computed voxel-wise t-statistics for the contrast between a given category and the average of all others. For each category, multivoxel patterns (MVPs) were defined as vectors of the corresponding t-value across all voxels within a given ROI (lateral VTC in the left or right hemisphere).

Correction: n/a

Models & analysis

n/a	Involved in the study
☒	☐ Functional and/or effective connectivity
☒	☐ Graph analysis
☐	☒ Multivariate modeling or predictive analysis

Multivariate modeling and predictive analysis: We used multivoxel patterns (MVPs) and representational similarity matrices (RSMs) to calculate face and word distinctiveness (see above) in right and left lateral ventral temporal cortex of each participant.
