# Supplementary material for: Active vision is linked to category selectivity in the individual brain
Source: Nat Hum Behav. 2026 Jul 7;10(9):1808–20. doi: 10.1038/s41562-026-02494-5 (PMC13590408; doi:10.1038/s41562-026-02494-5)
Supplement: Supplementary file 3 [file 41562_2026_2494_MOESM3_ESM.pdf]

nature portfolio

Peer Review File

Active vision is linked to category selectivity in the individual brain

Corresponding Author: Ms Diana Kollenda

Version 0:

Decision Letter:

29th September 2025

Dear Ms Kollenda,

Thank you once again for your manuscript, entitled "Active vision is tuned to representational distinctiveness in the individual brain", and for your patience during the peer review process.

Your Article has now been evaluated by 3 referees. You will see from their comments copied below that, although they find your work of potential interest, they have raised quite substantial concerns. In light of these comments, we cannot accept the manuscript for publication, but would be interested in considering a revised version if you are willing and able to fully address reviewer and editorial concerns.

We hope you will find the referees' comments useful as you decide how to proceed. If you wish to submit a substantially revised manuscript, please bear in mind that we will be reluctant to approach the referees again in the absence of major revisions. We are committed to providing a fair and constructive peer-review process. Do not hesitate to contact us if there are specific requests from the reviewers that you believe are technically impossible or unlikely to yield a meaningful outcome.

In particular, Reviewer #3 suggests performing a multivariate searchlight analysis. We recommend following this suggestion, as it would make the paper stronger. Reviewers #1 and #2 highlight issues with attention being a confounding factor and the role of hemispheric lateralization, which we ask you to address as well.

Finally, your revised manuscript must comply fully with our editorial policies and formatting requirements. Failure to do so will result in your manuscript being returned to you, which will delay its consideration. To assist you in this process, I have attached a checklist that lists all of our requirements. If you have any questions about any of our policies or formatting, please don't hesitate to contact me.

If you wish to submit a suitably revised manuscript, we would hope to receive it within 4 months. I would be grateful if you could contact us as soon as possible if you foresee difficulties with meeting this target resubmission date.

With your revision, please:

• Include a “Response to the editors and reviewers” document detailing, point-by-point, how you addressed each editor and referee comment. If no action was taken to address a point, you must provide a compelling argument. When formatting this document, please respond to each reviewer comment individually, including the full text of the reviewer comment verbatim followed by your response to the individual point. This response will be used by the editors to evaluate your revision and sent back to the reviewers along with the revised manuscript.

• Highlight all changes made to your manuscript or provide us with a version that tracks changes.

Please use the link below to submit your revised manuscript and related files:

Link Redacted

Note: This URL links to your confidential home page and associated information about manuscripts you may have submitted, or that you are reviewing for us. If you wish to forward this email to co-authors, please delete the link to your homepage.

Thank you for the opportunity to review your work. Please do not hesitate to contact me if you have any questions or would like to discuss the required revisions further.

Sincerely,

[redacted]
Nature Human Behaviour

Reviewer expertise:

Reviewer #1: Visual perception, fMRI, MVPA

Reviewer #2: Face perception, neuroimaging, pattern analysis

Reviewer #3: Vision, gaze, brain imaging, brain stimulation

REVIEWER COMMENTS:

Reviewer #1 (Remarks to the Author):

The main finding of this study is a correlation between individual gaze biases for faces and words and the representational distinctiveness of these categories in ventral temporal cortex (VTC), as measured with fMRI. The study additionally reports correlations between neural measures and performance on reading and face recognition tasks.

My overall evaluation of the study is very positive; the paper is easy to follow, the tasks are simple and validated, and the evidence is convincing. The interpretation of the findings is not quite so straightforward (especially teasing apart cause and effect) but the findings are interesting and relevant for understanding individual differences in visual cognition.

Comments and suggestions:
-In l.51-52, the authors cite a previous study that reported that fixation biases predict representational divergence in inferior temporal cortex, which sounds similar to the current findings. How was that study different from the current study?

-I feel that the authors could do more to consider (and to be explicit about) the two main interpretations of the correlation between individual gaze biases for faces and words and the representational distinctiveness of these categories in VTC: 1) neural distinctiveness causes gaze biases, e.g., by making objects more salient (this seems to be the preferred interpretation, though the text is somewhat vague about it); or 2) gaze biases (i.e., a person's visual diet) increase neural distinctiveness (without neural distinctiveness necessarily causing gaze biases during free viewing). These explanations are not mutually exclusive and they may work in tandem, but this is not necessarily the case (and future studies could test this). I found sentences describing the effect quite vague, e.g., that gaze is "tuned" to representational distinctiveness (l. 72-74; what does this mean?); why not just write that gaze is correlated with representational distinctiveness?

-A third (less interesting) interpretation is that participants with a face (word) bias paid more attention to the faces (words) in the fMRI experiment, resulting in more distinct representations (but this being task-specific). Similarly, participants with better face (word) recognition skills may have been able to better process the rapidly presented faces (words). Again, on this argument, the observed visual cortex distinctiveness may be task-specific, only arising in tasks with rapid presentation or (lack of) attentional demands for example. The authors could discuss this alternative interpretation, which has also featured in studies investigating the role of expertise in driving selectivity in VTC (e.g., Harel et al., Cerebral Cortex 2010).

-More generally, the authors could include a link to studies investigating expertise; for example, increased car recognition skills (i.e., expertise) increases selectivity (and presumably distinctiveness) in VTC (Gauthier et al., Nat Neuro 2000), increases car detection in scenes (Reeder et al., Psych Bull Rev 2016) and also increases the bottom-up salience of cars (Stein et al., JEP:HPP 2016). In other words, studies of expertise similarly support a link between neural distinctiveness in VTC, recognition skills, orienting in scenes, and bottom-up salience.

-I'm happy with the dependent measure of distinctiveness as defined here, but it would be useful for the field to know whether classic univariate measures of face-/word- selectivity show a similar relationship with gaze biases and recognition performance, considering that this is the measure that many studies (including developmental studies) have used. This could be reported in the Supp material. The authors may also want to consider whether hemispheric lateralization (faces-right vs words-left) predicts gaze biases (e.g., work by Behrmann).

-The main text selectively reports some correlations (sometimes with r- and p-value, sometimes only (non-significant) p-value). Please report all relevant correlations in tables in the Supp material. For example, on l.163: word distinctiveness did not correlate with gaze biases to faces; this is an important control/comparison as it rules out that some subjects were just better (e.g., compliant) in everything, but it is not clear whether this correlation goes in the same direction (and is similar in magnitude) as the correlation of interest. Also, l.183 reports the correlation between reading-speed scores and pseudowords, but not for words (and was the p-value corrected for these multiple comparisons?). I do believe that the main findings are robust, but the selective reporting of some correlations (out of potentially many) is not good practice – see also next point.

-It was unclear to me if/how tests of correlations were corrected for multiple comparisons. There is a sentence about this in the methods (l.500), but this is not clear when reading the main text; could you make explicit for each section what p-value would be considered significant (given the number of comparisons in that section)?

-Please provide more information about the oddball task performed in the scanner. For example, how many targets were there?

What was the performance and did performance in face/word blocks correlate, across individuals, with corresponding category-specific gaze biases (if so, that would be a concern)?

Reviewer #2 (Remarks to the Author):

This study aims to address individual differences of gaze patterns when viewing complex scenes, and how this relates to distinctiveness of visual category representations in the ventral temporal cortex (VTC). Over 100 adults were recruited for the eye-tracking experiment of viewing everyday scenes, and subsets also participated subsequent fMRI and behavioral tests to examine their VTC response patterns corresponding to visual stimuli of different categories, like faces or words, and their abilities in face recognition and reading. The results indicate that where individuals look in a scene (particularly for faces vs. text) reflects how distinctly their brain encodes those categories, and this in turn predicts how well they recognize faces or read.

This is an interesting study that provides valuable idiosyncratic data for addressing an important question in visual neuroscience. The manuscript is also clearly written; however, my concerns are listed below, ordered from relatively major to minor.

1. The results are only correlational. While important, I believe readers of Nature Human Behaviour would be more interested in mechanisms underlying active vision. It is not clear to me what the authors are ultimately arguing, except perhaps vaguely leaning towards the postulation that information processing in the VTC may guides oculomotor control. However, saccades toward faces are known to be able to occur very quickly, i.e., in astonishingly only 100 ms, whereas activity in the VTC is often associated with the N170, an ERP component that peaks around 170 ms and thus is presumably later than the initiation of fast saccades. Similarly, although I am less familiar with the literature on how quickly saccades toward texts or other categories can occur, the ventral visual pathway is generally thought to be a slower pathway for recognizing ‘what’ compared to the dorsal pathway for spatially localizing ‘where’. It is therefore unclear how such a slow ventral pathway could guide oculomotor control.

2. Attention mechanism may be a confounding factor. Could the term ‘active vision’ simply be replaced by attention? Attention, of course, guides fixation. Due to idiosyncratic attentional preferences, faces or text may attract more interests/attention for some individuals than others. It is not surprising that attention could sharpen representational distinctiveness in the VTC.

3. The hemispheric lateralization is interesting and could be elaborated or examined further. For example, why would face distinctiveness in the right lateral VTC correlate negatively with first fixations to text? Are left-lateralized text distinctiveness and right-lateralized face distinctiveness correlated? Do fixation patterns differ when faces/text were shown in the left vs. right visual field, and are such fixation differences correlated with hemispheric lateralization?

4. Several possible third variables may be worth considering. For example, is there any gender difference? Some previous studies have suggested a female advantage in overall face recognition. Is face representational distinctiveness stronger in female? Along similar lines, could personality traits such as extroversion vs. introversion be a factor? Are fixation patterns for text and text representational distinctiveness both correlated with performance in language/literature vs. STEM courses?

5. Is the idiosyncrasy of representational distinctiveness for faces and text simply due to larger or smaller fusiform face areas (FFA) and visual word form areas (VWFA) respectively? What about the surface area or cortical thickness of the VTC? Could structural idiosyncrasies of the VTC play a role here?

6. The manuscript lacks a statistical power analysis. Can the authors justify how they determined their sample size?

7. It is difficult to be certain whether participants truly had ‘normal or corrected-to-normal vision’. I assume the authors meant to say that participants had normal or corrected-to-normal visual acuity, with no known visual deficits as self-reported.

Reviewer #3 (Remarks to the Author):

This is an excellent study examining the relationship between individual propensities to fixate faces and text during viewing of natural images on the one hand and the distinctiveness of face- and text representations in individual brains. The authors find that increased multivariate distinctiveness measures of face- and text responses in ventral lateral cortex correlates with behavioural face- and text fixation measures. In addition, the authors provide behavioural analyses showing correlations between face recognition expertise and fixation properties during natural viewing.

Critique
Overall, the study is exceedingly clearly motivated, a pleasure to read, methodologically clean, and data are well-presented. I have no (true) major concerns or criticisms, except for two very minor suggestions and one optional major suggestion.

Major
The study relied heavily on previously defined, rather large brain areas (lateral ventral cortex), on which the complete analysis rested. This is fine, and well-motivated. Nevertheless, given that whole-brain imaging was performed: would it not be worthwhile to perform a multivariate searchlight analysis for distinctiveness measures and their correlation with behaviour? This would then complete the inference we can draw on this topic in terms of brain-behaviour relations. Alternatively, one could limit additional analyses to brain regions significantly responsive to faces and text vs scramble – which would undoubtedly include parietal and ventral prefrontal regions.

Minor:
Clarity:
Line 88: "Across 700 scenes, we found excellent split-half reliability (cf. de Haas et al. (2019)5 for the individual proportion of first fixations on faces" : at first reading of this, it's unclear if split-half refers to scenes or to participants. This becomes clear further down and in the figures, but it may help to clarify this at first-mention here.

Line 116: "low face distinctiveness" : same here: perhaps point the reader to expect a succinct summary of what "distinctiveness" means to improve the reading flow experience. E.g. "We excluded one participant as outlier due to extremely low face distinctiveness (see brief description below), .."

signed review: Andreas Bartels

Version 1:

Decision Letter:

Our ref: NATHUMBEHAV-25084006A

23rd March 2026

Dear Dr Kollenda,

Thank you for submitting your revised manuscript "Active vision is linked to category selectivity in the individual brain" (NATHUMBEHAV-25084006A). It has now been seen by the original referees and their comments are below. As you can see, the reviewers find that the paper has improved in revision. We will therefore be happy in principle to publish it in Nature Human Behaviour, pending minor revisions to comply with our editorial and formatting guidelines.

We are now performing detailed checks on your paper and will send you a checklist detailing our editorial and formatting requirements within two weeks. Please do not upload the final materials and make any revisions until you receive this additional information from us.

Please do not hesitate to contact me if you have any questions.

Sincerely,

[redacted]
Nature Human Behaviour

Reviewer #1 (Remarks to the Author):

The authors have addressed all my concerns.

Reviewer #2 (Remarks to the Author):

The authors have addressed all my previous concerns.

Reviewer #3 (Remarks to the Author):

The authors have addressed all my comments and suggestions in full. In addition, they clearly made a major and in my view successful effort to address a multitude of major and minor comments of additional referees. Overall this led to an improvement of the manuscript while the main conclusions remained. I hence fully support publication of the revised version.

Version 2:

Decision Letter:

Dear Ms Kollenda,

We are pleased to inform you that your Article "Active vision is linked to category selectivity in the individual brain", has now been accepted for publication in Nature Human Behaviour.

Authors may need to take specific actions to achieve compliance with funder and institutional open access mandates. If your research is supported by a funder that requires immediate open access (e.g. according to Plan S principles or the NIH public access policy) then you should select the gold OA route, and we will direct you to the compliant route where possible. Because authors warrant under our subscription licensing terms that they haven't committed to licensing any version of their article under a licence inconsistent with the terms of our agreement – including the applicable embargo period – publication under the subscription model isn't suitable for authors whose funders require no embargo.

Once your manuscript is typeset and you have completed the appropriate grant of rights, you will receive a link to your electronic proof via email with a request to make any corrections within 48 hours. If, when you receive your proof, you cannot meet this deadline, please inform us at rjsproduction@springernature.com immediately. Once your paper has been scheduled for online publication, the Nature press office will be in touch to confirm the details.

Acceptance of your manuscript is conditional on all authors' agreement with our publication policies (see http://www.nature.com/nathumbehav/info/gta). In particular your manuscript must not be published elsewhere and there must be no announcement of the work to any media outlet until the publication date (the day on which it is uploaded onto our web site).

If you have posted a preprint on any preprint server, please ensure that the preprint details are updated with a publication reference, including the DOI and a URL to the published version of the article on the journal website.

An online order form for reprints of your paper is available at https://www.nature.com/reprints/author-reprints.html. All co-authors, authors' institutions and authors' funding agencies can order reprints using the form appropriate to their geographical region.

We welcome the submission of potential cover material (including a short caption of around 40 words) related to your manuscript; suggestions should be sent to Nature Human Behaviour as electronic files (the image should be 300 dpi at 210 x 297 mm in either TIFF or JPEG format). Please note that such pictures should be selected more for their aesthetic appeal than for their scientific content, and that colour images work better than black and white or grayscale images. Please do not try to design a cover with the Nature Human Behaviour logo etc., and please do not submit composites of images related to your work. I am sure you will understand that we cannot make any promise as to whether any of your suggestions might be selected for the cover of the journal.

You can now use a single sign-on for all your accounts, view the status of all your manuscript submissions and reviews, access usage statistics for your published articles and download a record of your refereeing activity for the Nature journals.

To assist our authors in disseminating their research to the broader community, our SharedIt initiative provides you with a unique shareable link that will allow anyone (with or without a subscription) to read the published article. Recipients of the link with a subscription will also be able to download and print the PDF.

As soon as your article is published, you will receive an automated email with your shareable link.

In approximately 10 business days you will receive an email with a link to choose the appropriate publishing options for your paper and our Author Services team will be in touch regarding any additional information that may be required.

If you have any questions about our publishing options, costs, Open Access requirements, or our legal forms, please contact ASJournals@springernature.com

We look forward to publishing your paper.

With best regards,

[redacted]
Nature Human Behaviour

P.S. Click on the following link if you would like to recommend Nature Human Behaviour to your librarian http://www.nature.com/subscriptions/recommend.html#forms

** Visit the Springer Nature Editorial and Publishing website at www.springernature.com/editorial-and-publishing-jobs for more information about our career opportunities. If you have any questions please click here.**

Open Access This Peer Review File is licensed under a Creative Commons Attribution 4.0 International License, which permits use, sharing, adaptation, distribution and reproduction in any medium or format, as long as you give appropriate credit to the original author(s) and the source, provide a link to the Creative Commons license, and indicate if changes were made.
In cases where reviewers are anonymous, credit should be given to 'Anonymous Referee' and the source.
The images or other third party material in this Peer Review File are included in the article's Creative Commons license, unless indicated otherwise in a credit line to the material. If material is not included in the article's Creative Commons license and your intended use is not permitted by statutory regulation or exceeds the permitted use, you will need to obtain permission directly from the copyright holder.
To view a copy of this license, visit https://creativecommons.org/licenses/by/4.0/

Point-by-Point Response to the Reviewers

We thank the editor and reviewers for their comments, which we reply to in full below. Our responses to the reviewers' comments are indicated in blue under each comment. We have highlighted changes in the manuscript in yellow.

Additionally, we would like to point out that the data of participant ID52 was previously excluded as an outlier (as reported), but now is included in all analysis. We have identified the underlying problem as a misaligned anatomical mask, which we now corrected.

Additionally, we modified the title to "Active vision is linked to category selectivity in the individual brain". This reflects suggestions by the reviewers and corresponding new results, showing that the link between individual gaze tendencies and neural tuning includes, but is not limited to the more specific concept of distinctiveness.

Reviewer #1:

The main finding of this study is a correlation between individual gaze biases for faces and words and the representational distinctiveness of these categories in ventral temporal cortex (VTC), as measured with fMRI. The study additionally reports correlations between neural measures and performance on reading and face recognition tasks.

My overall evaluation of the study is very positive; the paper is easy to follow, the tasks are simple and validated, and the evidence is convincing. The interpretation of the findings is not quite so straightforward (especially teasing apart cause and effect) but the findings are interesting and relevant for understanding individual differences in visual cognition.

We thank the reviewer and will address the following comments and suggestions below.

Comments and suggestions:

-In l.51-52, the authors cite a previous study that reported that fixation biases predict representational divergence in inferior temporal cortex, which sounds similar to the current findings. How was that study different from the current study?

We appreciate the reviewer's question. The cited study by Borovska & de Haas, 2025, PNAS) and our work addressed different questions. Borovska and de Haas found that fixating different parts of the same movie leads to diverging responses in inferior temporal cortex. That is, individual gaze leads to systematic differences in retinal input, which yield concurrent representational differences. Here, we asked: Is individual gaze linked to differences in the visual processing of *identical retinal input*, that is, to processing differences as such? If so, *how* do people with different gaze differ in their neural representations? We found that localiser stimuli presented at central fixation are processed more distinctively in the brains of people who tend to look at stimuli from this category when embedded in complex scenes. In other words, individual gaze is systematically linked to category-dependent differences in the processing of identical retinal input. We fully agree that this should become clearer in the manuscript and now elaborate on this difference in the discussion section, lines 362-373:

"A previous study from our group demonstrated that individual gaze during movie-watching goes along with concurrent representational differences in the ventral stream. Specifically, the authors showed that the interindividual similarity of neural representations during movie watching is determined by the similarity of gaze[16]. Here, we asked different questions: Do neural representations *as such* differ between people with different gaze tendencies, even under conditions of central fixation? If so, *how* do neural representations differ between people with different gaze tendencies? We found that centrally presented faces and pseudowords are represented more distinctively in individuals who tend to fixate these objects when they are embedded in complex scenes. Moreover, the brains of individuals prioritizing faces or text had larger corresponding regions of interest in the ventral stream (rFFA and lVWFA, respectively). This suggests that gaze prioritizes retinal input which the individual brain can process more distinctively."

-I feel that the authors could do more to consider (and to be explicit about) the two main interpretations of the correlation between individual gaze biases for faces and words and the representational distinctiveness of these categories in VTC: 1) neural distinctiveness causes gaze biases, e.g., by making objects more salient (this seems to be the preferred interpretation, though the text is somewhat vague about it); or 2) gaze biases (i.e., a person's visual diet) increase neural distinctiveness (without neural distinctiveness necessarily causing gaze biases during free viewing). These explanations are not mutually exclusive and they may work in tandem, but this is not necessarily the case (and future studies could test this). I found sentences describing the effect quite vague, e.g., that gaze is "tuned" to representational distinctiveness (l. 72-74; what does this mean?); why not just write that gaze is correlated with representational distinctiveness?

We thank the reviewer for raising this point. We agree it's best to be explicit about the different potential interpretations and clearly differentiate what can be said about them at this point from what needs to be done in the future. We also changed the term "tuned" to the more neutral "is linked to" in the title, abstract (lines 27 and 34) and Result section headers and Supplementary Information, Table S1.

The corresponding manuscript changes can be found in the introduction and discussion sections:

Lines 73-80: "These parallel developmental trajectories raise the possibility of a systematic link between active vision and representational spaces. Specifically, gaze tendencies may follow neural development, actively prioritizing semantic categories which can be represented more distinctively. Vice versa, developing gaze tendencies and changes in visual diet may shape representational distinctiveness. These hypotheses are not mutually exclusive and share a straightforward prediction: If there is a systematic link between gaze and representational distinctiveness, large individual differences in gaze tendencies among adults should correlate with corresponding category distinctiveness in the individual brain."

Lines 406-440: "Why do active vision and neural tuning in the ventral stream match each other? Our main approach is correlational, but several additional observations lead us to an informed speculation: Individual gaze and neural tuning in the ventral stream may mutually shape each other across the first two decades of life, while adults match their moment-by-moment gaze to stabilized tuning properties of the mature ventral stream.

The development of both, gaze tendencies[25] and neural tuning in the ventral stream[23,26,47] is protracted across the first two decades of life. Developing fixation habits likely shape the individual foveal diet. Visual experience in turn can shape category-specific neural responses in the ventral stream, as shown for reading acquisition (e.g. [27,29,48-50]), extensive exposure to Pokémon characters[51,52] or face-deprivation in non-human primates[22]. At the same time, a lack of neural preferences goes along with a strongly reduced tendency to spontaneously fixate faces[21,22], underscoring the possibility of a bidirectional link. Moreover, spontaneous gaze shows surprisingly strong genetic components[10,11], which could partly be mediated by heritable differences in neural tuning[53,54], including ventral stream preferences for faces[55,56]. Taken together, individual differences in gaze and neural tuning may mutually reinforce each other across development. However, this remains to be tested in multimodal longitudinal studies (also see below).

In adults, both the individual layout of the ventral stream[57] and individual differences in gaze[5] appear stable. However, this stability seems more pronounced for neural tuning than gaze. While attention and task instructions can modulate ventral stream responses top-down, this does not lead to a reversal of category-specific preferences in areas like the FFA[58]. Spontaneous gaze tendencies, on the other hand, can be overridden and reversed top-down: Even individuals with a strong tendency to look at faces manage to mostly look away from them if instructed to do so[43]. This suggests that, at least for adults, the moment-by-moment plasticity of gaze exceeds that of neural tuning in the ventral stream. Furthermore, individual differences in adult neural response patterns do not depend on concurrent differences in gaze. The results of our localizer experiment documented such differences under conditions of central fixation. Taken together, adults appear to show persistent individual differences in their neural preferences, which gaze caters to second by second. Future studies should test the causal nature of this link.

More generally, further research should trace neural and gaze development longitudinally to disentangle their interplay. Doing this in mono- and dizygotic twins would allow to differentiate between genetic and environmental effects[10,11]. Furthermore, studies utilizing perceptual training could manipulate the distinctiveness of individual representations to test their effect on gaze."

-A third (less interesting) interpretation is that participants with a face (word) bias paid more attention to the faces (words) in the fMRI experiment, resulting in more distinct representations (but this being task-specific). Similarly, participants with better face (word) recognition skills may have been able to better process the rapidly presented faces (words). Again, on this argument, the observed visual cortex distinctiveness may be task-specific, only arising in tasks with rapid presentation or (lack of) attentional demands for example. The authors could discuss this alternative interpretation, which has also featured in studies investigating the role of expertise in driving selectivity in VTC (e.g., Harel et al., Cerebral Cortex 2010).

We agree that habitual differences in category-specific levels of attention would be a plausible (and in our view, interesting) mediator between gaze tendencies and category distinctiveness. Note that such attentional differences would have to generalise between centrally presented localiser stimuli, reading and face recognition tasks as well as the free viewing of complex scenes. Also note that the correlations

we observed are between tasks operating at different time scales, from saccadic behaviour, which requires several decisions per second to sustained neural responses in a block design and a face recognition task with self-paced trials (unlimited time).

To test for such general effects of attention, we conducted a new experiment, as detailed in lines 620-646 (Methods): "60 naïve participants (mean age = 22.87 years, SD = 3.25; 8 male and 52 female) completed a shorter, but otherwise similar version of the free-viewing experiment reported above (using 200 instead of 700 images; cf. Linka & de Haas, 2020[7]). Again, calibration was accepted only if the maximum error was ≤ 1.2 degrees of visual angle (dva) and the average error was ≤ 0.6 dva. The observed mean validation error was 0.35 dva (SD = 0.08 dva). These participants also completed a dual-task based on the localizer experiment. The task lasted ~10 min and contained 6 practice trials and 84 experimental trials total (2 x 42, alternating face and pseudowords). The experimental trials were divided into three equal blocks (28 trials each) with short breaks in between. The stimuli (spanning 28 dva square) and oddball task were identical to the face and pseudoword blocks in the localizer experiment, except for the addition of a secondary task. For the primary task, participants used the left arrow key to report the oddball target of a phase-scrambled background (i.e. no object overlayed; same task as in the scanner). The new, secondary task ("blob detector") added a small (4.3 dva), mid-grey disk in one of the four image corners on 1-3 frames per trial. These secondary targets were reported using the right arrow key and considered a hit if they occurred within 1 s of onset. Per category, the blob counts followed a fixed distribution (3 trials with 1 blob occurence, 20 with 2, 19 with 3), yielding 200 secondary targets overall (100 during face trials, 100 during word trials). Image order and blob positions were identical for all participants (fixed assignment), and the minimum temporal spacing between any two targets (primary or secondary) was two primary stimuli (606 ms during face trials, 1000 ms during word trials). Timing and keypresses were logged continuously. For each participant, we computed category-specific hit rates (e.g., number of secondary targets in face blocks/hits) and the difference in hit rate between text and face blocks (text – face; that is, positive and negative values indicate an advantage during the face and text blocks, respectively). To assess split-half reliability, we computed this indicator and category-specific hit rates separately for the first and second halves of the experiment and correlated it across participants."

As well as in the result section (lines 280-300): "The link between gaze tendencies and category-selective activity in VTC could in principle be mediated by category-specific levels of attention, generalizing between free-viewing and the localizer task. Specifically, participants with a stronger tendency to fixate faces or words during free-viewing may also have allocated higher levels of attention to the respective type of stimulus when it was presented centrally and in isolation during the fMRI localizer task. This hypothesis would align with the finding that experts direct more attention to their object of expertise[64-66], potentially leading to increased activation levels in VTC[67-70].

To test this hypothesis, we conducted an additional psychophysics experiment in 60 naïve adults who also completed an abbreviated, but sensitive version of the free-viewing experiment (200 images; cf. Linka & de Haas, 2020[7]). Crucially, the new psychophysics experiment mirrored our fMRI localizer design but introduced a

secondary blob-detection task to probe differential levels of attentional competition with faces or pseudowords (cf.[71], and see Methods for details). This design enabled two key tests: First, to determine whether there are systematic individual differences in the level of attentional competition afforded by isolated, centrally presented faces and pseudowords. Second, to test whether these differences in attention correlate with the tendency to fixate faces or words during free-viewing. While a positive correlation would support the mediation account, the absence of a detectable correlation would speak against attentional engagement mediating the observed link between gaze tendencies and category-selective activity in VTC."

The results of this experiment confirmed systematic individual differences in attentional load for faces and words (and their differential). However, these differences in attentional load were unrelated to systematic differences in gaze tendencies, as now reported in lines 301-310:

"First, participants indeed showed reliable individual differences in secondary task performance for face blocks (M = 73%, SD = 10.2%; split-half reliability r = .66, p < .001), pseudoword blocks (M = 85%, SD = 17.2%; split-half reliability r = .92, p < .001), and the performance difference between them (face-block advantage M = 11.7% SD = 16.6%; split-half reliability r = .81, p <. 001). Second, however, we found no significant associations between this indicator of category-specific differential levels of attention and gaze tendencies towards faces (first fixations: r = -.03, p = .814, dwell time: r = .08, p = .528) or text (first fixations: r = -.03, p = .844, dwell time: r = -.01, p = .951). This suggests that the relationship between gaze and category-selective activity in VTC is unlikely to be mediated by elevated levels of category-specific attention."

As discussed in lines 441-449: "Theoretically, both gaze tendencies and neural preferences could be mediated by habitually elevated levels of covert and overt attention. If an individual has persistently elevated levels of attention for a given type of stimulus, this may boost corresponding neural responses and bias gaze in tandem. If true, this should also render the corresponding stimulus a more potent distractor in dual task scenarios. Our additional psychophysical attention experiment was designed to put this prediction to a well-powered test. However, we found no such relationship, rendering persistent general differences in category-specific levels of attention an unlikely mediating mechanism for the relationship between neural tuning and gaze."

We address the role of expertise separately below.

Regarding potential task specificity, we think a limitation to rapid processing is highly unlikely, given the correlations with the self-paced Cambridge Face Memory Test in our current study and in a previous sample (de Haas et al., 2019, *PNAS*), as well as systematically increased levels of face salience in super-recognisers (Linka et al., 2022, *Sci Rep*). Moreover, as outlined in the methods, stimulus frequencies in the localiser task were chosen to maximise category-specific responses, in line with previous results (Stigliani et al., 2015, *J. Neurosci*). Nevertheless, we agree that future studies should test the generalisability of individual differences, neural distinctiveness and perceptual skills across tasks, ideally including real world behaviour. We have now added this point to the comparison with other expertise effects in the discussion (see below).

-More generally, the authors could include a link to studies investigating expertise; for example, increased car recognition skills (i.e., expertise) increases selectivity (and presumably distinctiveness) in VTC (Gauthier et al., Nat Neuro 2000), increases car detection in scenes (Reeder et al., Psych Bull Rev 2016) and also increases the bottom-up salience of cars (Stein et al., JEP:HPP 2016). In other words, studies of expertise similarly support a link between neural distinctiveness in VTC, recognition skills, orienting in scenes, and bottom-up salience.

We thank the reviewer for highlighting this and now discuss our findings in relation to these previous studies, lines 450-465: "Notably, expertise may play a role for both, salience and cortical selectivity (cf. [51,52]). For instance, greater car expertise is associated with better car detection in scenes[66], increased bottom-up salience for cars[72], and increased VTC selectivity[67]. Given performance differences on face recognition and reading tasks go along with corresponding category selectivity and gaze tendencies, our results can be interpreted along these lines. Developmentally, gaze tendencies, perceptual skills and neural tuning may reinforce each other (see above). This could be interpreted as a spiral of expertise, achieving different levels in different individuals.

Interestingly, however, expertise research found that widespread category-selective activity for objects of expertise diminished to novice-like responses when stimulus engagement was low[70]. Future studies could test whether high category-specific engagement tasks increase the size of the effects we observed here. This could be part of a broader endeavour to test how well individual differences in gaze, perceptual skills and neural tuning generalise between different types of tasks, including real-world behaviour. A first step in this direction is the observation of elevated face salience in super recognisers, some of whom are employed for real-world face recognition tasks by the police[9]."

-I'm happy with the dependent measure of distinctiveness as defined here, but it would be useful for the field to know whether classic univariate measures of face-/word- selectivity show a similar relationship with gaze biases and recognition performance, considering that this is the measure that many studies (including developmental studies) have used. This could be reported in the Supp material. The authors may also want to consider whether hemispheric lateralization (faces-right vs words-left) predicts gaze biases (e.g., work by Behrmann).

We agree and now have added tests of a potential relation between gaze tendencies and more "classic" univariate indices of category selectivity on the one hand and hemispheric lateralization on the other.

First, we computed univariate measures of face and word-selectivity by quantifying participant-specific selective activation within group-level parcels for the right fusiform face area (rFFA) and left visual word form area (lVWFA; as detailed in Methods, section "Definition of functional ROIs and their individual size", lines 697-716). The results reported in the results section 'The individual sizes of rFFA and lVWFA are linked to gaze tendencies and behavioural performance' (lines 219-278). In summary, our findings confirmed high split-half reliability for functional region of interest sizes

across odd and even functional runs. Furthermore, we observed significant category-specific correlations: the size of rFFA was positively associated with both, the initial fixation bias toward faces and behavioural performance on the CFMT. Conversely, the tendency to first fixate text correlated positively with the size of lVWFA.

We have now added the Methods section titled "Definition of functional ROIs and their individual size", lines 697-716:
"We defined functional regions of interest (fROIs) using the automated Group-constrained Subject-Specific (GSS) approach (cf. 76-78). This method provides unbiased, participant-specific fROIs and is highly suitable for our sample size (N = 61). First, individual 1st-Level GLMs were estimated in MNI space using unsmoothed data. We then used the spm_ss toolbox (https://www.nitrc.org/projects/spm_ss; 78) to generate binary activation maps for the contrasts of faces > other categories and words > other categories, respectively, both thresholded at $p < .0001$ (uncorrected). These binary maps were overlaid across participants, spatially smoothed (FWHM = 6mm), and thresholded to retain only voxels with at least 10% overlap across the sample. Resulting group-level parcels were restricted to those within which at least 60% (36/61) of participants showed significant activation. We compared resulting group-level peaks of respective parcels to previous literature and focused on the right Fusiform Face Area (rFFA, MNI peak coordinates: +40, -47, -21; cf. 76) and left Visual Word Form Area (lVWFA; MNI peak coordinates: -44, -66, -13; cf. 50; see Figure 4a, for an illustration of fROI locations).

To quantify participant-specific activation, we performed a masked voxel-count analysis. Each participant's t-contrast map (unsmoothed data; faces > other categories or words > other categories) was masked by the respective group-level parcel (rFFA or lVWFA). Within these constraints, we calculated the total number of significant voxels exceeding a threshold of $p < .0001$ (uncorrected; see Figure 4a and b)."

The results are included in a new subsection, "The individual sizes of rFFA and lVWFA are linked to gaze tendencies and behavioural performance" (lines 219-278):

"Our previous analyses focused on the multivariate measure of category distinctiveness within anatomically defined ROIs, which develops in tandem to category-specific salience (cf. [25,26]). However, many previous studies employed univariate measures of face and word-selectivity (cf. [23,29,30]). Everything else staying equal, both types of measures are expected to scale with the signal to noise ratio of category-specific responses. However, in principle they may also vary independently. While distinctiveness probes the sharpness of (potentially distributed) category-specific response patterns at the population level, the extent of category-specific responses probes the amount of neural resources dedicated to processing a given category. Here, we sought to test whether individual gaze tendencies and perceptual performance are also linked to univariate category selectivity, specifically the individual sizes of the right fusiform face (rFFA) and left visual word form area (lVWFA).

To this end, we quantified the participant-specific selective activation within group-level parcels for the rFFA and lVWFA (cf. Julian et al., 2012[76]; see methods for

details). We observed high split-half reliability (odd versus even localizer runs) for inter-individual differences in the sizes of these functionally defined regions of interest (rFFA: $r = .92$, $p < .001$; lVWFA: $r = .82$, $p < .001$). See Supplementary Table S1 for an overview of all tested correlations with functional region of interest sizes.

Individual sizes of rFFA correlated positively with the tendency to first fixate on faces ($r = .39$, $p = .002$) and showed a trend for the tendency of higher dwell times on faces ($r = .28$, $p = .031$, n.s. at the Bonferroni corrected threshold of $\alpha = .025$). In contrast, individual rFFA sizes were negatively correlated with gaze tendencies towards text (first fixations: $r = -37$, $p = .004$; dwell time: $r = -.34$, $p = .007$, surviving Bonferroni correction). Furthermore, rFFA sizes correlated positively with face distinctiveness in the right lateral VTC ($r = .41$, $p < .001$) and with behavioural performance on the CFMT ($N = 34$; $r = .37$, one-tailed $p = .017$; cf. Methods and Figure 4b). The latter result aligns with previous findings by Furl et al. (2011)[14] and Elbich & Scherf (2017)[15].

Individual sizes of lVWFA correlated positively with the tendency to first fixate text ($r = .35$, $p = .006$) and showed a corresponding trend for dwell time on text ($r = .26$, $p = .039$, n.s. at the Bonferroni corrected threshold of $\alpha = .025$). No significant cross-correlations were observed for gaze tendencies towards faces (first fixations: $r = .09$, $p = .514$; dwell time: $r = .01$, $p = .962$). lVWFA sizes showed a strong positive correlation with word distinctiveness in the left lateral VTC ($r = .75$, $p < .001$). However, the correlation between lVWFA sizes and reading-speed scores for pseudowords did not reach significance ($N = 25$; $r = .32$, one-tailed $p = .060$; cf. Methods, Figure 4c and Table S1), which may reflect the small sample size, especially in light of previous reports of significant correlations between lVWFA size and reading scores[37].

Overall, these results mirror the patterns observed between gaze tendencies, category distinctiveness and behaviour (cf. Figure 3 and Supplementary Table S1). They suggest that active vision is linked to both: the sharpness of neural tuning for and resources dedicated to processing a given category in the individual brain. Our results are also consistent with the previous developmental finding that increases in category selectivity go along with increases in category distinctiveness (cf. [26]). However, in contrast to distinctiveness, category-specific associations between areas sizes and dwell time as well as reading scores were limited to non-significant statistical trends."

Furthermore, we computed hemispheric laterality indices for face-word selective responses in VTC, as now detailed in Supplementary Methods, section "Calculation of fMRI laterality index per category", and results reported in the Supplementary Information, section 'No robust correlations between lateralization of category-specific responses and gaze tendencies or behavioural performance'. In sum, participants varied substantially in the degree of category laterality but showed the expected pattern at the group level: face-selective activation was on average right lateralized and word-selective activation was left lateralized. However, across participants, we found no strong evidence for relationships between laterality indices and gaze tendencies or behavioural performance.

-The main text selectively reports some correlations (sometimes with r- and p-value, sometimes only (non-significant) p-value). Please report all relevant correlations in tables in the Supp material. For example, on l.163: word distinctiveness did not correlate with gaze biases to faces; this is an important control/comparison as it rules out that some subjects were just better (e.g., compliant) in everything, but it is not clear whether this correlation goes in the same direction (and is similar in magnitude) as the correlation of interest. Also, l.183 reports the correlation between reading-speed scores and pseudowords, but not for words (and was the p-value corrected for these multiple comparisons?). I do believe that the main findings are robust, but the selective reporting of some correlations (out of potentially many) is not good practice – see also next point.

We thank the reviewer for raising this point. We now refer in the Results section to Table S1, which lists all correlations. For example, here we report the not significant cross-correlations between word distinctiveness in left lateral VTC and (1) first fixations and (2) dwell time on faces:

(1) $r = .16$, $p = .222$
(2) $r = -.03$, $p = .818$

Including the following note on multiple comparisons: "*Note.* * indicates significant correlations. The significance level was Bonferroni-corrected to account for the use of two salience metrics (proportion of first fixations and dwell time), and two speed reading scores (words and pseudowords). Adjusted significance levels and test directionality are denoted for each comparison." We elaborate on this approach below.

-It was unclear to me if/how tests of correlations were corrected for multiple comparisons. There is a sentence about this in the methods (l.500), but this is not clear when reading the main text; could you make explicit for each section what p-value would be considered significant (given the number of comparisons in that section)?

We appreciate this comment and have clarified our approach in the revised main text (e.g. lines 156-161), including the methods section (lines 731-755):

"We tested two primary a priori hypotheses: First, individual face salience is linked to face distinctiveness in the right lateral VTC and second, individual text salience is linked to word distinctiveness in the left lateral VTC. We quantified category-specific individual salience as the individual proportions of first fixations and dwell time spent on corresponding stimuli during the free-viewing experiment. To maintain statistical rigor, we adjusted significance thresholds for the use of these two metrics using the method of Bonferroni. We report original P-values but only interpret values below $\alpha = .025$ (.05/2) as significant. Note, we signalled significant results with asterisks in Table S1. We also adopted a conservative stance by employing two-sided tests, despite directional predictions for positive associations. We also tested the hypotheses that individual perceptual performance for reading and face recognition are linked to respective individual fixation tendencies and to corresponding representational distinctiveness. Here, we also corrected for the two salience measures tested (where applicable), but employed one-sided tests. This decision was based on previous

findings supporting the directional hypotheses (cf. [14,15,26]) and the lower sample sizes for perceptual performance tests. Significance levels for associations with speed reading scores were additionally corrected for the two reading measures employed (pseudowords and words).

Complementing our main analyses, we explored associations across hemispheres and categories (e.g. face distinctiveness in left lateral VTC vs. face salience and face distinctiveness in right lateral VTC vs. text salience). These served as specificity controls to test if the observed relationships between neural and gaze patterns are hemisphere- and category-specific. Following reviewer suggestions, we also explored associations with lVWFA and rFFA sizes. For all these exploratory analyses, we applied the same corrections as for the main analysis to account for the two metrics of salience (proportion of first fixations and dwell time)."

-Please provide more information about the oddball task performed in the scanner. For example, how many targets were there? What was the performance and did performance in face/word blocks correlate, across individuals, with corresponding category-specific gaze biases (if so, that would be a concern)?

We now updated the Methods section 'Localizer experiment' to provide more detailed information about the oddball task, including statistics on task performance, which was not related to individual gaze tendencies or category selectivity (lines 589-600): "Each run consisted of six blocks of each category. These blocks were shown in random order and intermixed with rest blocks showing only a grey background. Category blocks contained 12 – 27 stimuli, whereas rest blocks contained no stimuli. Participants were instructed to view the images while fixating on a central dot throughout. In each category block, one target was randomly interspersed, consisting of only the phase-scrambled background (i.e. no object overlayed). The oddball task required participants to press a button within 1 s of the appearance of this target. Responses from two participants were excluded due to a button-box malfunction. For the remaining 59 participants, performance on the oddball task during scanning was high for both face (hit rate = 80%, SD = 15%) and word blocks (hit rate = 83%, SD = 13%). In a supplementary analysis, we found no correlations between performance in the oddball tasks and category-specific gaze tendencies or category distinctiveness (see Table S2)."

Reviewer #2 (Remarks to the Author):

This study aims to address individual differences of gaze patterns when viewing complex scenes, and how this relates to distinctiveness of visual category representations in the ventral temporal cortex (VTC). Over 100 adults were recruited for the eye-tracking experiment of viewing everyday scenes, and subsets also participated subsequent fMRI and behavioral tests to examine their VTC response patterns corresponding to visual stimuli of different categories, like faces or words, and their abilities in face recognition and reading. The results indicate that where individuals look in a scene (particularly for faces vs. text)

reflects how distinctly their brain encodes those categories, and this in turn predicts how well they recognize faces or read.

This is an interesting study that provides valuable idiosyncratic data for addressing an important question in visual neuroscience. The manuscript is also clearly written; however, my concerns are listed below, ordered from relatively major to minor.

> We thank the reviewer for their thorough and thoughtful critique. Our point-by-point responses to their comments are provided below.

1. The results are only correlational. While important, I believe readers of Nature Human Behaviour would be more interested in mechanisms underlying active vision. It is not clear to me what the authors are ultimately arguing, except perhaps vaguely leaning towards the postulation that information processing in the VTC may guides oculomotor control. However, saccades toward faces are known to be able to occur very quickly, i.e., in astonishingly only 100 ms, whereas activity in the VTC is often associated with the N170, an ERP component that peaks around 170 ms and thus is presumably later than the initiation of fast saccades. Similarly, although I am less familiar with the literature on how quickly saccades toward texts or other categories can occur, the ventral visual pathway is generally thought to be a slower pathway for recognizing 'what' compared to the dorsal pathway for spatially localizing 'where'. It is therefore unclear how such a slow ventral pathway could guide oculomotor control.

> We thank the reviewer for raising this point. However, we would like to point out that direct neural recordings show VTC responses covering a range of latencies, including pre-saccadic category-selective responses. In non-human primates, face-selective responses have been documented below 100 ms (e.g., Issa & Di Carlo, 2012, *J. Neurosci*), as well as pre-saccadically for scene-embedded search (Sheinberg & Logothetis, 2001, *J. Neurosci*). Moreover, preliminary reports of eCOG data confirm pre-saccadic category-specific VTC responses in humans (Alreja et al., 2025, *JoV*). This renders a role of VTC in guiding fixation behaviour plausible. At the same time, we note that individual differences in ultra-rapid saccades towards faces are only weakly correlated with corresponding gaze tendencies during free-viewing (Broda et al., 2024, *JoV*), highlighting the possibility of multiple underlying mechanisms operating at different time-scales.
>
> More generally, our results reveal that moment-by-moment fixation behaviour caters to neural tuning in the individual ventral stream. We agree that our results do not provide final answers on *how* this is achieved by the visual system. We consider this one of the promising avenues for future research opened up by our findings. In our view this underscore rather than impedes their relevance. We have now expanded our discussion of possible neural mechanisms (lines 386 - 395):
>
> "Notably, Vinken & Vogels (2019)[21] reported a pronounced reduction of face salience for early fixations in a monkey with lacking face patches. Furthermore, Sheinberg & Logothetis (2001)[40] found that neurons in the temporal cortex of monkeys exhibited selective activation shortly before saccades to behaviourally relevant (i.e., target) objects, and this activity raised again during fixation. Similarly, a preliminary report of intracranial recordings confirms pre-saccadic, category-specific VTC responses in humans[44]. Thus, although VTC traditionally is not considered part of the oculomotor

system, it appears tightly linked to the control of gaze, perhaps by signalling object identity and relevance to downstream visuomotor networks (cf. [41,42]). Future research is needed to clarify the nature and timing of this potential influence."

2. Attention mechanism may be a confounding factor. Could the term 'active vision' simply be replaced by attention? Attention, of course, guides fixation. Due to idiosyncratic attentional preferences, faces or text may attract more interests/attention for some individuals than others. It is not surprising that attention could sharpen representational distinctiveness in the VTC.

We agree with the reviewer that generally elevated levels of category-specific attention appear a plausible mediating mechanism for the relationship we found. We consider this an interesting empirical question. To test this hypothesis, we conducted a new study with 60 naïve adults who participated in both a free-viewing experiment and a psychophysics experiment, which mirrored our fMRI localizer design but introduced a secondary task to test levels of attentional competition in face and pseudoword blocks (also see above, reviewer 1):

To test for such general effects of attention, we conducted a new experiment, as detailed in lines 619-646 (Methods): "60 naïve participants (mean age = 22.87 years, SD = 3.25; 8 male and 52 female) completed a shorter, but otherwise similar version of the free-viewing experiment reported above (using 200 instead of 700 images; cf. Linka & de Haas, 2020[7]). Again, calibration was accepted only if the maximum error was ≤ 1.2 degrees of visual angle (dva) and the average error was ≤ 0.6 dva. The observed mean validation error was 0.35 dva (SD = 0.08 dva). These participants also completed a dual-task based on the localizer experiment. The task lasted ~ 10 min and contained 6 practice trials and 84 experimental trials total (2 x 42, alternating face and pseudowords). The experimental trials were divided into three equal blocks (28 trials each) with short breaks in between. The stimuli (spanning 28 dva square) and oddball task were identical to the face and pseudoword blocks in the localizer experiment, except for the addition of a secondary task. For the primary task, participants used the left arrow key to report the oddball target of a phase-scrambled background (i.e. no object overlayed; same task as in the scanner). The new, secondary task ("blob detector") added a small (4.3 dva), mid-grey disk in one of the four image corners on 1-3 frames per trial. These secondary targets were reported using the right arrow key and considered a hit if they occurred within 1 s of onset. Per category, the blob counts followed a fixed distribution (3 trials with 1 blob occurence, 20 with 2, 19 with 3), yielding 200 secondary targets overall (100 during face trials, 100 during word trials). Image order and blob positions were identical for all participants (fixed assignment), and the minimum temporal spacing between any two targets (primary or secondary) was two primary stimuli (606 ms during face trials, 1000 ms during word trials). Timing and keypresses were logged continuously. For each participant, we computed category-specific hit rates (e.g., number of secondary targets in face blocks/hits) and the difference in hit rate between text and face blocks (text – face; that is, positive and negative values indicate an advantage during the face and text blocks, respectively). To assess split-half reliability, we computed this indicator and category-specific hit rates separately for the first and second halves of the experiment and correlated it across participants."

As well as in the result section (lines 280-300): "The link between gaze tendencies and category-selective activity in VTC could in principle be mediated by category-specific levels of attention, generalizing between free-viewing and the localizer task. Specifically, participants with a stronger tendency to fixate faces or words during free-viewing may also have allocated higher levels of attention to the respective type of stimulus when it was presented centrally and in isolation during the fMRI localizer task. This hypothesis would align with the finding that experts direct more attention to their object of expertise[64-66], potentially leading to increased activation levels in VTC[67-70].

To test this hypothesis, we conducted an additional psychophysics experiment in 60 naïve adults who also completed an abbreviated, but sensitive version of the free-viewing experiment (200 images; cf. Linka & de Haas, 2020[7]). Crucially, the new psychophysics experiment mirrored our fMRI localizer design but introduced a secondary blob-detection task to probe differential levels of attentional competition with faces or pseudowords (cf.[71], and see Methods for details). This design enabled two key tests: First, to determine whether there are systematic individual differences in the level of attentional competition afforded by isolated, centrally presented faces and pseudowords. Second, to test whether these differences in attention correlate with the tendency to fixate faces or words during free-viewing. While a positive correlation would support the mediation account, the absence of a detectable correlation would speak against attentional engagement mediating the observed link between gaze tendencies and category-selective activity in VTC."

The results of this experiment confirmed systematic individual differences in attentional load for faces and words (and their differential). However, these differences in attentional load were unrelated to systematic differences in gaze tendencies, as now reported in lines 301-310:

"First, participants indeed showed reliable individual differences in secondary task performance for face blocks (M = 73%, SD = 10.2%; split-half reliability r = .66, p < .001), pseudoword blocks (M = 85%, SD = 17.2%; split-half reliability r = .92, p < .001), and the performance difference between them (face-block advantage M = 11.7% SD = 16.6%; split-half reliability r = .81, p <. 001). Second, however, we found no significant associations between this indicator of category-specific differential levels of attention and gaze tendencies towards faces (first fixations: r = -.03, p = .814, dwell time: r = .08, p = .528) or text (first fixations: r = -.03, p = .844, dwell time: r = -.01, p = .951). This suggests that the relationship between gaze and category-selective activity in VTC is unlikely to be mediated by elevated levels of category-specific attention."

As discussed in lines 441-449: "Theoretically, both gaze tendencies and neural preferences could be mediated by habitually elevated levels of covert and overt attention. If an individual has persistently elevated levels of attention for a given type of stimulus, this may boost corresponding neural responses and bias gaze in tandem. If true, this should also render the corresponding stimulus a more potent distractor in dual task scenarios. Our additional psychophysical attention experiment was designed to put this prediction to a well-powered test. However, we found no such relationship, rendering persistent general differences in category-specific levels of

attention an unlikely mediating mechanism for the relationship between neural tuning and gaze."

Thus, while attention is closely coupled with eye movements and can modulate responses in VTC in principle, we found no evidence that the relationship between gaze tendencies and category distinctiveness is mediated by generally elevated levels of category-specific attention. Therefore, we think it is appropriate to stick to the more specific and descriptive term 'active vision'.

3. The hemispheric lateralization is interesting and could be elaborated or examined further. For example, why would face distinctiveness in the right lateral VTC correlate negatively with first fixations to text? Are left-lateralized text distinctiveness and right-lateralized face distinctiveness correlated? Do fixation patterns differ when faces/text were shown in the left vs. right visual field, and are such fixation differences correlated with hemispheric lateralization?

These are interesting questions, which we now investigated. However, we did not find any robust relationships between category-specific laterality and gaze tendencies or category distinctiveness, as now reported in the Supplementary Information, section 'No robust correlations between lateralization of category-specific responses and gaze tendencies or behavioural performance'.

We also examined whether the degree of left spatial dominance in first fixations for faces or text relates to hemispheric lateralization for faces or text. However, we observed no significant associations (faces: $r = .03$, $p = .809$; text: $r = -.02$, $p = .899$).

4. Several possible third variables may be worth considering. For example, is there any gender difference? Some previous studies have suggested a female advantage in overall face recognition. Is face representational distinctiveness stronger in female? Along similar lines, could personality traits such as extroversion vs. introversion be a factor? Are fixation patterns for text and text representational distinctiveness both correlated with performance in language/literature vs. STEM courses?

We appreciate these suggestions and address them in turn.

Gender: While several studies reported a visual preference for social stimuli in females (e.g. Lutchmaya & Baron-Cohen, 2002, *Infant Behavior and Development*; Proverbio, 2017, *Journal of neuroscience research*; Proverbio et al., 2008, BMC neuroscience), a recent large-scale study reported only small differences and in the opposite direction (Linka et al., 2025, *NHB*): males looked slightly more at faces, females slightly more at text (d = -.06 dwell time on text; d = .23 for dwell time on faces and d = .16 for first fixations on faces). Notably, this large-scale study had a sample size orders of magnitude higher than previous studies (>7,000) and documented that gender differences are dwarfed by *individual* differences, casting a cautionary light on conclusions of general gender differences based on small samples. Given this and our unbalanced sample structure (19 male; 42 female), any gender analysis would be underpowered.

Personality (extraversion/introversion): In principle, this could be an interesting candidate moderator, but existing work has found no clear links between the gaze

tendencies we tested and such personality measures (e.g. de Haas et al., 2019, *PNAS*).

Academic performance (language/literature vs. STEM): This seems an interesting avenue for future research, especially given the association with reading scores.

We think that these points are worth discussing in the manuscript and now included them in lines 478-487: "Larger and more diverse samples could probe the role of multiple third variables and potential moderators such as gender, personality traits or academic-domain performance. However, the evidence for gender and personality associations with gaze tendencies is limited. For example, some previous studies observed a visual preference for social stimuli in females[73-75], while recent large-scale eye-tracking work hast reported only very small gender differences in the opposite direction (Linka et al., 2025[25]; d ≈ 0.2). Likewise, prior work has found no reliable link between personality traits like extraversion and gaze tendencies (de Haas et al., 2019[5]). Given the observed associations with reading scores, it seems especially important to investigate the developmental interplay of schooling, gaze tendencies, neural development and educational attainment."

5. Is the idiosyncrasy of representational distinctiveness for faces and text simply due to larger or smaller fusiform face areas (FFA) and visual word form areas (VWFA) respectively? What about the surface area or cortical thickness of the VTC? Could structural idiosyncrasies of the VTC play a role here?

We thank the reviewer for raising these points. In principle, both category distinctiveness and the size of functional regions above threshold should scale with the signal-to-noise ratio of category selective responses. However, these measures address distinct biological questions and their answers can vary independently (also see below): While the size of FFA and VWFA reflects the amount of neural resources dedicated to category-specific processing, distinctiveness reflects the sharpness of (potentially distributed) category-specific responses at the population level. Whether individual gaze is linked to both, category-specific distinctiveness and the size of corresponding regions therefore is an interesting (non-redundant) empirical question. We have now added tests of a potential relation between gaze tendencies and individual sizes of FFA and VWFA .

First, we computed univariate measures of face and word-selectivity by quantifying participant-specific selective activation within group-level parcels for the right-lateralized FFA and left-lateralized VWFA (as detailed in Methods, section "Definition of functional ROIs and their individual size", lines 697-716, see below). The results are reported in the results section 'The individual sizes of rFFA and lVWFA are linked to gaze tendencies and behavioural performance' (lines 219-278, including Figure 4, see below). In summary, our findings confirmed high split-half reliability for functional region sizes across odd and even functional runs. Furthermore, we observed significant category-specific correlations: the size of rFFA was positively associated with both, the tendency to first fixate on faces and behavioural performance on the CFMT. Conversely, the tendency to first fixate text correlated positively with the size of lVWFA.

Methods, "Definition of functional ROIs and their individual size", lines 697-716:

“We defined functional regions of interest (fROIs) using the automated Group-constrained Subject-Specific (GSS) approach (cf. 76-78). This method provides unbiased, participant-specific fROIs and is highly suitable for our sample size (N = 61). First, individual 1st-Level GLMs were estimated in MNI space using unsmoothed data. We then used the spm_ss toolbox (https://www.nitrc.org/projects/spm_ss; 78) to generate binary activation maps for the contrasts of faces > other categories and words > other categories, respectively, both thresholded at $p < .0001$ (uncorrected). These binary maps were overlaid across participants, spatially smoothed (FWHM = 6mm), and thresholded to retain only voxels with at least 10% overlap across the sample. Resulting group-level parcels were restricted to those within which at least 60% (36/61) of participants showed significant activation. We compared resulting group-level peaks of respective parcels to previous literature and focused on the right Fusiform Face Area (rFFA, MNI peak coordinates: +40, -47, -21; cf. 76) and left Visual Word Form Area (lVWFA; MNI peak coordinates: -44, -66, -13; cf. 50; see Figure 4 a, for an illustration of fROI locations).

To quantify participant-specific activation, we performed a masked voxel-count analysis. Each participant’s t-contrast map (unsmoothed data; faces > other categories or words > other categories) was masked by the respective group-level parcel (rFFA or lVWFA). Within these constraints, we calculated the total number of significant voxels exceeding a threshold of $p < .0001$ (uncorrected; see Figure 4a and b).”

The results are included in a new subsection, “The individual sizes of rFFA and lVWFA are linked to gaze tendencies and behavioural performance” (lines 219-278):

“Our previous analyses focused on the multivariate measure of category distinctiveness within anatomically defined ROIs, which develops in tandem to category-specific salience (cf. [25,26]). However, many previous studies employed univariate measures of face and word-selectivity (cf. [23,29,30]). Everything else staying equal, both types of measures are expected to scale with the signal to noise ratio of category-specific responses. However, in principle they may also vary independently. While distinctiveness probes the sharpness of (potentially distributed) category-specific response patterns at the population level, the extent of category-specific responses probes the amount of neural resources dedicated to processing a given category. Here, we sought to test whether individual gaze tendencies and perceptual performance are also linked to univariate category selectivity, specifically the individual sizes of the right fusiform face (rFFA) and left visual word form area (lVWFA).

To this end, we quantified the participant-specific selective activation within group-level parcels for the rFFA and lVWFA (cf. Julian et al., 2012[76]; see methods for details). We observed high split-half reliability (odd versus even localizer runs) for inter-individual differences in the sizes of these functionally defined regions of interest (rFFA: $r = .92$, $p < .001$; lVWFA: $r = .82$, $p < .001$). See Supplementary Table S1 for an overview of all tested correlations with functional region of interest sizes.

Individual sizes of rFFA correlated positively with the tendency to first fixate on faces ($r = .39$, $p = .002$) and showed a trend for the tendency of higher dwell times on faces

(r = .28, p = .031, n.s. at the Bonferroni corrected threshold of α = .025). In contrast, individual rFFA sizes were negatively correlated with gaze tendencies towards text (first fixations: r = -37, p = .004; dwell time: r = -.34, p = .007, surviving Bonferroni correction). Furthermore, rFFA sizes correlated positively with face distinctiveness in the right lateral VTC (r = .41, p < .001) and with behavioural performance on the CFMT (N = 34; r = .37, one-tailed p = .017; cf. Methods and Figure 4b). The latter result aligns with previous findings by Furl et al. (2011)[14] and Elbich & Scherf (2017)[15].

Individual sizes of lVWFA correlated positively with the tendency to first fixate text (r = .35, p = .006) and showed a corresponding trend for dwell time on text (r = .26, p = .039, n.s. at the Bonferroni corrected threshold of α = .025). No significant cross-correlations were observed for gaze tendencies towards faces (first fixations: r = .09, p = .514; dwell time: r = .01, p = .962). lVWFA sizes showed a strong positive correlation with word distinctiveness in the left lateral VTC (r = .75, p < .001). However, the correlation between lVWFA sizes and reading-speed scores for pseudowords did not reach significance (N = 25; r = .32, one-tailed p = .060; cf. Methods, Figure 4c and Table S1), which may reflect the small sample size, especially in light of previous reports of significant correlations between lVWFA size and reading scores[37].

Overall, these results mirror the patterns observed between gaze tendencies, category distinctiveness and behaviour (cf. Figure 3 and Supplementary Table S1). They suggest that active vision is linked to both: the sharpness of neural tuning for and resources dedicated to processing a given category in the individual brain. Our results are also consistent with the previous developmental finding that increases in category selectivity go along with increases in category distinctiveness (cf. [26]). However, in contrast to distinctiveness, category-specific associations between areas sizes and dwell time as well as reading scores were limited to non-significant statistical trends."

Figure 4: The relationship between the individual size of rFFA and lVWFA, gaze tendencies and behavioural performance.

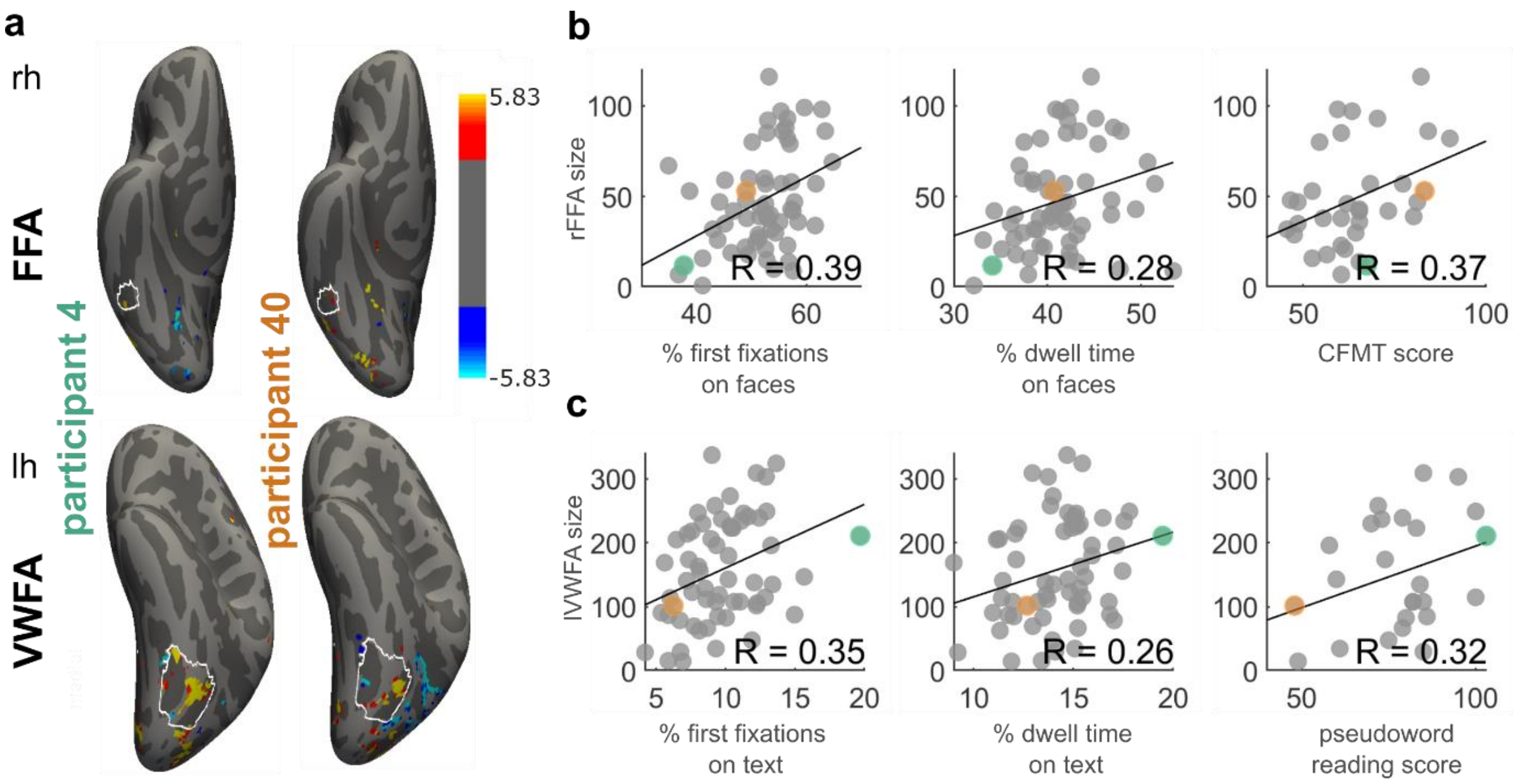

(a) the top panel shows the outline of the group-level rFFA parcel in MNI space overlaid with individual t-contrasts for face-specific activations, ranging from |t| > 4.28 (p < .0001) to 5.83, as indicated by the colour bar to the right. Similarly, the bottom panel shows group-level parcels for the lVWFA overlaid with individual t-contrasts for word-specific activations. (b) Correlations between the individual size of rFFA and the proportion of first fixations on faces (N = 61, p = .002), the proportion of dwell time on faces (N = 61, p = .031, n.s., Bonferroni-corrected), and the Cambridge Face Memory Test (CFMT) score (N = 34, one-tailed p = .014), respectively. (c) Correlations between the individual size of lVWFA and the proportion of first fixations on text (N = 61, p = .006), the proportion of dwell time on text (N = 61, p = .039, n.s., Bonferroni-corrected), and the pseudoword reading score (N = 25, one-tailed p = .060, n.s.), respectively. Coloured dots highlight the same individuals across figures (participant ID4 in green, participant ID40 in orange).

Regarding the surface area (mm²) or average cortical thickness (mm) of the right and left lateral VTC, structural idiosyncrasies were not associated with category distinctiveness nor with gaze tendencies (see the following table for an overview).

**Test**	**Result**
Correlation between surface area of left lateral VTC and word distinctiveness in the left lateral VTC	r = -.08, p = .456
Correlation between surface area of right lateral VTC and face distinctiveness in the right lateral VTC	r = -.11, p = .409
Correlation between or average cortical thickness of left lateral VTC and word distinctiveness in the left lateral VTC	r = -.20, p = .120
Correlation between or average cortical thickness of right lateral VTC and face distinctiveness in the left lateral VTC	r = -.11, p = .375
Correlation between surface area of left lateral VTC and (1) first fixations and (2) dwell time on text	*(1)* r = .16, p = .228<br>*(2)* r = .13, p = .301

Correlation between surface area of right lateral VTC and (1) first fixations and (2) dwell time on faces	*(1)* $r = -.11$, $p = .375$<br>*(2)* $r = -.11$, $p = .375$
Correlation between average cortical thickness of left lateral VTC and (1) first fixations and (2) dwell time on text	*(1)* $r = .01$, $p = .950$<br>*(2)* $r = -.10$, $p = .438$
Correlation between average cortical thickness of right lateral VTC and (1) first fixations and (2) dwell time on faces	*(1)* $r = .17$, $p = .184$<br>*(2)* $r = .21$, $p = .106$

6. The manuscript lacks a statistical power analysis. Can the authors justify how they determined their sample size?

> We now specify this in the reporting summary and Methods section (lines 756-765): "Our sample size aimed for good sensitivity (power > .85) for effect sizes of r >= .4. This was based on previously observed effect sizes for the relation between category-specific distinctiveness and perceptual skills among children[26]. Under our hypothesis, we expected somewhat larger effect sizes for the relationship between representational distinctiveness and gaze tendencies among adults, given our estimates of distinctiveness were based on three times the number of localizer runs per data point (six instead of two; Nordt et al, 2023[26]); the generally better signal-to-noise ratio of BOLD responses in adults (e.g. Thomason et al., 2005[79]); and the excellent internal consistency of individual differences in face- and word-fixation tendencies among adults[5,80]."

7. It is difficult to be certain whether participants truly had 'normal or corrected-to-normal vision'. I assume the authors meant to say that participants had normal or corrected-to-normal visual acuity, with no known visual deficits as self-reported.

> We thank the reviewer for pointing this out and revised the sentence in the Participants section to the more precise: "All participants had normal or corrected-to-normal visual acuity and reported no known visual deficits." (lines 501-502)

Reviewer #3 (Remarks to the Author):

This is an excellent study examining the relationship between individual propensities to fixate faces and text during viewing of natural images on the one hand and the distinctiveness of face- and text representations in individual brains. The authors find that increased multivariate distinctiveness measures of face- and text responses in ventral lateral cortex correlates with behavioural face- and text fixation measures. In addition, the authors provide behavioural analyses showing correlations between face recognition expertise and fixation properties during natural viewing.

Critique

Overall, the study is exceedingly clearly motivated, a pleasure to read, methodologically clean, and data are well-presented. I have no (true) major concerns or criticisms, except for two very minor suggestions and one optional major suggestion.

We appreciate the reviewer's constructive feedback and have addressed his specific comments and suggestions in the responses below.

Major

The study relied heavily on previously defined, rather large brain areas (lateral ventral cortex), on which the complete analysis rested. This is fine, and well-motivated. Nevertheless, given that whole-brain imaging was performed: would it not be worthwhile to perform a multivariate searchlight analysis for distinctiveness measures and their correlation with behaviour? This would then complete the inference we can draw on this topic in terms of brain-behaviour relations. Alternatively, one could limit additional analyses to brain regions significantly responsive to faces and text vs scramble – which would undoubtedly include parietal and ventral prefrontal regions.

We appreciate this suggestion and have now included a searchlight analysis for the category distinctiveness measures, as detailed in the Supplementary Methods and Results ('Exploratory searchlight analysis' and 'Whole brain searchlight analysis of category distinctiveness and its relation to gaze tendencies'). Briefly, this analysis revealed that large parts of the inferior temporal cortex and parietal areas show distinctive category representations (Figures S1a and S2a). Furthermore, this analysis reproduced the finding that ventral temporal cortex (VTC) distinctiveness is related to gaze tendencies, specifically between word distinctiveness and the tendency of first fixations on text, and face distinctiveness and dwell time on faces. Additionally, the tendency to direct first fixations towards text correlated with word distinctiveness in areas of the extended reading network. These findings are now referenced in the main Results section (lines 173-177):

"An exploratory whole-brain searchlight analysis replicated associations between gaze tendencies and corresponding category distinctiveness in VTC. Moreover, we found that the tendency to direct first fixations to text correlated with word distinctiveness in regions of the extended reading network, especially in the left hemisphere (cf. [62,63]; see Supplementary Methods and Results, including Figures S1 and S2)."

Minor:

Clarity:

Line 88: "Across 700 scenes, we found excellent split-half reliability (cf. de Haas et al. (2019)5 for the individual proportion of first fixations on faces" : at first reading of this, it's unclear if split-half refers to scenes or to participants. This becomes clear further down and in the figures, but it may help to clarify this at first-mention here.

Thank you for pointing this out. We agree that the original phrasing was ambiguous and revised the sentence to: "For the individual proportion of first fixations on faces, we found excellent split-half reliability across odd and even images, r = .93, p <.001 (maximum-to-minimum ratio of 2.04)." (lines 91-92)

Line 116: “low face distinctiveness” : same here: perhaps point the reader to expect a succinct summary of what “distinctiveness” means to improve the reading flow experience. E.g. “We excluded one participant as outlier due to extremely low face distinctiveness (see brief description below), ..”

> Thank you for this suggestion. However, we now recovered the corrupted anatomical ROI mask for this participant and now could include their data, rendering this sentence obsolete.

signed review: Andreas Bartels

Point-by-point response

Reviewer #1 (Remarks to the Author):

The authors have addressed all my concerns.

Reviewer #2 (Remarks to the Author):

The authors have addressed all my previous concerns.

Reviewer #3 (Remarks to the Author):

The authors have addressed all my comments and suggestions in full. In addition, they clearly made a major and in my view successful effort to address a multitude of major and minor comments of additional referees. Overall this led to an improvement of the manuscript while the main conclusions remained. I hence fully support publication of the revised version.

We thank all reviewers for their help in getting the paper to this stage!
